# Integrating Targeted Metabolomics and Targeted Proteomics to Study the Responses of Wheat Plants to Engineered Nanomaterials

**DOI:** 10.1021/acsagscitech.4c00046

**Published:** 2024-04-02

**Authors:** Weiwei Li, Arturo A. Keller

**Affiliations:** Bren School of Environmental Science and Management, University of California at Santa Barbara, Santa Barbara, California 93106, United States

**Keywords:** engineered nanomaterials (ENMs), targeted metabolomics, targeted proteomics, multiomics, joint-pathway analysis

## Abstract

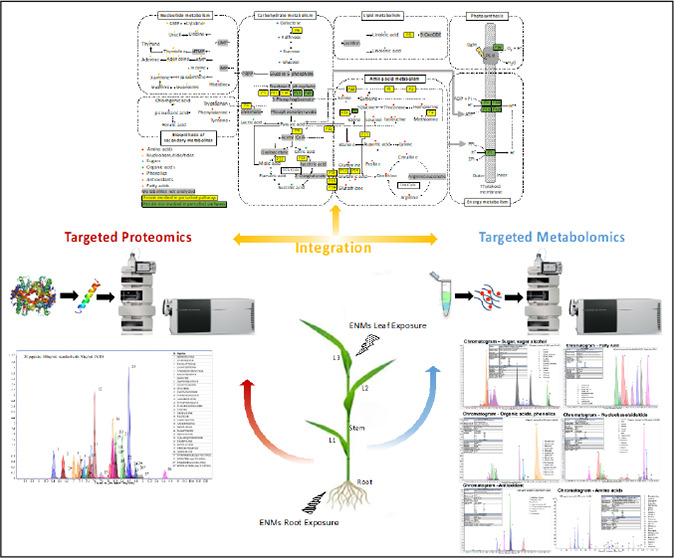

This manuscript presents a multiomics investigation into the metabolic and proteomic responses of wheat to molybdenum (Mo)- and copper (Cu)-based engineered nanomaterials (ENMs) exposure via root and leaf application methods. Wheat plants underwent a four-week growth period with a 16 h photoperiod (light intensity set at 150 μmol·m^–2^·s^–1^), at 22 °C and 60% humidity. Six distinct treatments were applied, including control conditions alongside exposure to Mo- and Cu-based ENMs through both root and leaf routes. The exposure dosage amounted to 6.25 mg of the respective element per plant. An additional treatment with a lower dose (0.6 mg Mo/plant) of Mo ENM exclusively through the root system was introduced upon the detection of phytotoxicity. Utilizing LC–MS/MS analysis, 82 metabolites across various classes and 24 proteins were assessed in different plant tissues (roots, stems, leaves) under diverse treatments. The investigation identified 58 responsive metabolites and 19 responsive proteins for Cu treatments, 71 responsive metabolites, and 24 responsive proteins for Mo treatments, mostly through leaf exposure for Cu and root exposure for Mo. Distinct tissue-specific preferences for metabolite accumulation were revealed, highlighting the prevalence of organic acids and fatty acids in stem or root tissues, while sugars and amino acids were abundant in leaves, mirroring their roles in energy storage and photosynthesis. Joint-pathway analysis was conducted and unveiled 23 perturbed pathways across treatments. Among these, Mo exposure via roots impacted all identified pathways, whereas exposure via leaf affected 15 pathways, underscoring the reliance on exposure route of metabolic and proteomic responses. The coordinated response observed in protein and metabolite concentrations, particularly in amino acids, highlighted a dynamic and interconnected proteomic-to-metabolic-to-proteomic relationship. Furthermore, the contrasting expression patterns observed in glutamate dehydrogenase (upregulation at 1.38 ≤ FC ≤ 1.63 with high Mo dose, and downregulation at 0.13 ≤ FC ≤ 0.54 with low Mo dose) and its consequential impact on glutamine expression (7.67 ≤ FC ≤ 39.60 with high Mo dose and 1.50 ≤ FC ≤ 1.95 with low Mo dose) following Mo root exposure highlighted dose-dependent regulatory trends influencing proteins and metabolites. These findings offer a multidimensional understanding of plant responses to ENMs exposure, guiding agricultural practices and environmental safety protocols while advancing knowledge on nanomaterial impacts on plant biology.

## 1 Introduction

Engineered nanomaterials (ENMs) have emerged as significant elements in agriculture in the past decade, notably as nanopesticides and nanofertilizers, aiming to augment agricultural productivity and sustainability.^1−4^ This is crucial in meeting the challenges posed by feeding an expanding global population (exceeding 10 billion in 35 years) amidst the backdrop of climate change.^5^ Due to its ability to deliver active ingredients precisely and employ controlled release mechanisms, nanotechnology represents a potential solution to address the evolving agricultural demands.^4^ It offers innovative methods to enhance the crop yield and plant resilience in the face of environmental stressors. Thus, gaining a deeper understanding of how these nanomaterials interact with biological systems at the cellular level is crucial to developing safer and more efficient applications in agricultural practices. Especially, owing to the rapid analytical improvements in liquid chromatography–mass spectrometry (LC–MS), targeted analytical approaches enable tissue-specific analysis, to provide more detailed understanding of the effects of ENMs on particular plant tissues.^6^ This level of analysis contributes to refining the design and application of ENMs in agriculture to ensure their effectiveness while concurrently mitigating potential risks or adverse impacts on plants, soil, and the surrounding environment.

Understanding the intricate mechanisms governing cellular responses to varying environmental stimuli, including ENMs treatments, is pivotal to unraveling the complexity of biological systems. The advent of high-throughput technologies in various “omics” fields such as genomics, transcriptomics, proteomics, and metabolomics has significantly expanded our capacity to explore biological systems at different molecular levels.^7,8^ In general, genomics (genes level) provides collective characterization and quantification of the organism’s genes, while transcriptomics (mRNA level) looks into gene expression patterns determined by RNA transcript. Proteomics (proteins level) studies dynamic protein products and their interactions, while metabolomics (metabolites level) profiles metabolites, the final downstream product of gene expression, at a specific time under specific environmental conditions. When a plant is exposed to any xenobiotic, the processes triggered are interconnected, involving gene expression regulation, subsequent protein regulation, and alterations in metabolic processes that ultimately manifest in the plant’s phenotype. Integrating these data sets through multiomics can serve to comprehensively understand the complex interactions and regulatory networks within biological systems.^9,10^ For example, by integrating metabolomics and transcriptomics, a study revealed the regulation of the genes in the flavonoid biosynthesis pathway that promoted the biosynthesis of quinone chalcones in safflower under MeJA treatment.^11^ Another study integrated proteome and metabolome profiling with alterations in the levels of enzymes of glycolysis and TCA cycle pathways and relative metabolites revealed protein profiling and metabolism disturbances induced by the differential transformation process in glyphosate tolerant genetically modified maize.^12^

The existing multiomics investigations have primarily employed untargeted approaches, enabling a broad and comprehensive view at each level of omics analysis. However, untargeted approaches have limitations in terms of accuracy, sensitivity, and reproducibility compared to targeted methods, which focus on specific molecules or pathways of interest.^13−16^ Thus, in our study, we opted to utilize our previous optimized targeted metabolomics^17^ and targeted proteomics^18^ approaches. We aimed to investigate the specific molecular responses of plants to ENMs at both the protein and metabolite levels. This strategic approach allows us to focus on particular molecules or pathways of interest, providing a more precise and detailed understanding of how plants respond to ENM exposure. Furthermore, through the utilization of targeted omics analytical techniques, researchers can transcend static snapshots and explore the temporal dynamics of molecular responses. This enhanced methodology can enrich our comprehension of intricate biological processes, offering insights into the kinetics, dynamics, and adaptability of organisms under varying environmental or experimental circumstances.

In this study, we focused on wheat (*Triticum aestivum*), a globally significant crop, to investigate the impact of two types of ENMs, specifically molybdenum (Mo)-based nanofertilizer and copper (Cu)-based nanopesticide. We investigated two exposure routes: root exposure and leaf exposure since they represent two common application approaches in agriculture. This investigation aids in understanding the potentially different effects and responses of plants to ENMs administered through different application techniques.^6^ We selected 24 proteins for analysis based on previous research indicating their susceptibility to perturbation upon exposure to ENMs.^18^ A total of 82 metabolites that were actively involved in plant central metabolism were selected for targeted metabolomics analysis, including antioxidants, organic acids, phenolics, nucleobase/side/tide, amino acids, sugar/sugar alcohol, and fatty acids.^17,19,20^ The significance of our research lies in the potential for guiding agricultural practices and environmental safety protocols by providing a comprehensive understanding of how plants respond to exposure to ENMs. By taking into account ENM design, dose optimization, and exposure routes, this project aims to contribute to the advancement of sustainable agricultural practices and facilitate the utilization of nanotechnology’s benefits while mitigating potential risks to plants, ecosystems, and human health.

## 2 Materials and Methods

### 2.1 Characteristics of ENMs

Cu(OH)_2_-NMs (99.5% purity, diameter 50 nm, length 3–5 μm, US3078) and MoO_3_-NMs (99.94% purity, average particle size 13–80 nm, US3330) were purchased from U.S. Research Nanomaterials Inc. (Houston, TX, USA). ENM suspensions were freshly prepared by sonication for 30 min and applied to wheat as ENM treatments through two exposure routes, root and leaf. For root exposure, ENM suspensions containing Cu or Mo (1250 mg of element/L) were prepared in 10% Hoagland solution. On day 7, instead of regular watering, Cu and Mo exposure groups were watered with ENMs suspensions (25 mg of Cu or Mo per pot) evenly distributed in pots to ensure root exposure. For leaf exposure, ENM suspensions containing Cu or Mo (500 mg element/L) were prepared in a surfactant solution (0.2% Triton X-100 in NANOpure water). From day 22 to day 28, plant leaves were soaked 3 times daily in ENM suspensions to receive 7 mL/day for exposure groups or in surfactant solution for the leaf control group. The total ENM exposure for both root and leaf exposure routes was 6.25 mg of Cu or Mo per plant (25 mg per pot). At least 40 plant replicates were raised for each treatment group in both exposure approaches. In addition to the existing treatment levels, an extra lower concentration of 0.6 mg of Mo per plant was introduced via the roots. This lower concentration was included in the experiment to evaluate the recommended field-application dose of Mo. The selection of all dosage levels, including this lower concentration, was based on findings and recommendations from prior studies to ensure a comprehensive assessment of Mo.^6,19,21−23^

In a prior study, we examined the dissolution rates of Cu- and Mo-based ENMs.^24^ Cu ENMs dissolved slowly, around 1% in both DI water and root exudate solution over 6 days, at a rate of 0.001% per hour. Mo ENMs dissolved rapidly, releasing 31–35% of Mo ions within the first 6 h and 0.026% to 0.047% per hour afterward. Consequently, wheat plants exposed to Mo ENMs via roots or leaves would be significantly exposed to Mo^6+^, while exposure to Cu ENMs would result in low concentrations of Cu^2+^ in either case. Despite the potential insights that non-nanoscale controls could provide in distinguishing the effects of nanoparticles from those of elemental or ionic forms, we prioritized the inclusion of nanoscale treatments due to our main focus on demonstrating the effectiveness of multiomics approaches with nanoscale agrochemicals.

### 2.2 Wheat Growth and Harvest

*Triticum aestivum* (wheat) seeds purchased from Harmony Farms KS (Jennings, KS, USA) were sterilized using a 1% sodium hypochlorite solution for 10 min followed by rinsing with NANOpure water and soaking in NANOpure water overnight before germination. Vermiculite saturated with 10% Hoagland water was prepared and transferred into plant pots to serve as soil. Soaked seeds (four seeds per pot) were planted in the soil with their tips facing up to ensure successful germination. Each pot was watered daily with 20 mL of 10% Hoagland water to maintain adequate moisture. Plants were grown under specific conditions: 16 h photoperiod, light intensity of 150 μmol·m^–2^·s^–1^, temperature of 22 °C, and 60% relative humidity for 4 weeks. In total, 6 treatment groups, including root exposure control, Cu exposure through root, Mo exposure through root, leaf exposure control, Cu exposure through leaf, and Mo exposure through leaf, were harvested on day 28. Three leaves emerged from each plant during the 4-week growth period. The harvested plants were cut into 5 parts, including leaf #1 (L1), leaf #2 (L2), leaf #3 (L3), stem, and root, with L1 being the first leaf to emerge and L3 the third leaf to emerge. The pooled tissue of each part was homogenized using mortar and pestle coupled with liquid nitrogen, and then stored in 50 mL centrifuge tubes at −80 °C until analyzed.

### 2.3 Metabolites Extraction and Targeted Metabolomics Analysis

To extract metabolites from harvested plants, a universal extraction method from our previous studies was used.^17^ Generally, a portion of 100 mg of plant tissue from each homogenized part was mixed with 1 mL of 80% methanol in water with 2% formic acid in a 1.5 mL centrifuge tube by vortexing at 3000 rpm for 20 min, followed by sonication in a water bath for 20 min at room temperature. Then, the extraction was centrifuged at 20,000*g* for 20 min, and the 1 mL of supernatant was divided and transferred into 4 vials with 200 μL in each, followed by reconstitution into proper solvent for liquid chromatography with tandem mass spectrometry (LC–MS/MS) analysis grouped by 6 metabolite categories, including antioxidants (vial #1), organic acids and phenolics (vial #2), nucleobase/side/tides (vial #2), amino acids (vial #3), sugar/sugar alcohol (vial #3), and fatty acids (vial #4) (Figure S1). A full list of metabolites analyzed using our targeted metabolomics analysis is presented in Table S1, detailing the information on reconstitution solvent, optimized LC–MS/MS column, and mobile phase. The LC–MS/MS analysis parameters for targeted metabolomics analysis are detailed in Table S2. LC–MS/MS chromatographs of the 6 groups of metabolites using optimized methods are shown in Figure S2. For quality assurance and quality control (QA/QC) purposes, a midlevel calibration standard was injected following every 6 sample injections, accompanied by a solvent blank. The recovery rates for QC injections consistently fell within the range of 80% to 120%.

### 2.4 Protein Extraction and Targeted Proteomics Analysis

Tissue samples were processed using a phenol extraction method coupled with trypsin digestion.^6,18^ Generally, 200 mg of plant tissue was extracted using a phenol extraction buffer and partitioned with a Tris-buffered phenol solution. Then, protein was precipitated using 0.1 M ammonium acetate in methanol overnight at −20 °C. The protein pellet was solubilized in 8 M urea with 50 mM ammonium bicarbonate solution, followed by reduction with 5 mM DTT, alkylation with 20 mM IAA, and digestion with 2 μg of trypsin enzyme overnight at 37 °C with rotation. The digested peptides were purified using a C-18 solid-phase extraction cartridge and finally reconstituted in 30% acetonitrile in water with 5% formic acid and 3% DMSO for LC–MS/MS analysis. Based on our previous study,^6^ 24 proteins were selected and analyzed using targeted proteomics (Table S3). The peptide analysis was conducted using an Agilent Polaris 3 C18-Ether column (150 × 3.0 mm, p/n: A2021150X030) coupled with a gradient mobile phase system (A: water + 0.1% (v:v) formic acid + 3% (v:v) DMSO; B: ACN + 0.1% (v:v) formic acid + 3% (v:v) DMSO) developed in our previous studies.^6 ,18^ A needle wash with TFE was added between injections to reduce the carryover. To ensure QA/QC, a midlevel calibration standard was injected after every 6 sample injections, alongside a solvent blank. The recovery rates for QC injections consistently ranged between 80% and 120%.^6^

### 2.5 Statistical Analysis and Integrated Pathway Analysis

Partial least squares-discriminant analysis (PLS-DA) was employed to visualize the separation between different treatment groups.^25^ Volcano plots were used to illustrate the relationship between fold changes in metabolites expression and statistical significance (represented by negative logarithm of *p*-values), to help in pinpointing responsive metabolites and proteins with significant changes.^11,26^ Heatmaps were utilized to display metabolite abundance across different treatments, enabling identification of clusters of metabolites with similar expression profiles and highlighting differences or trends among experimental groups. Fold change bar plots were generated to prioritize metabolites that exhibit substantial changes with magnitudes larger than 25%. In addition, Venn diagrams were used for visualizing overlaps and differences between different treatment groups. Pathway analysis was performed with identified responsive metabolites and proteins for each treatment using MetaboAnalyst 5.0 coupled with the KEGG pathway library. The threshold of impact value calculated from pathway topology analysis (relative-betweenness centrality) was set at 0.1 for the identification of perturbed pathways.^17,19^ Moreover, a pathway mapping based on KEGG templates was created with responsive metabolites and proteins involved in perturbed pathways to provide a comprehensive visual representation, demonstrating the interplay among responsive metabolites, proteins, and the perturbed pathways and exploring their relationships across different omics layers.

## 3 Results and Discussion

### 3.1 Targeted Metabolites Analysis

**Figure 1:**
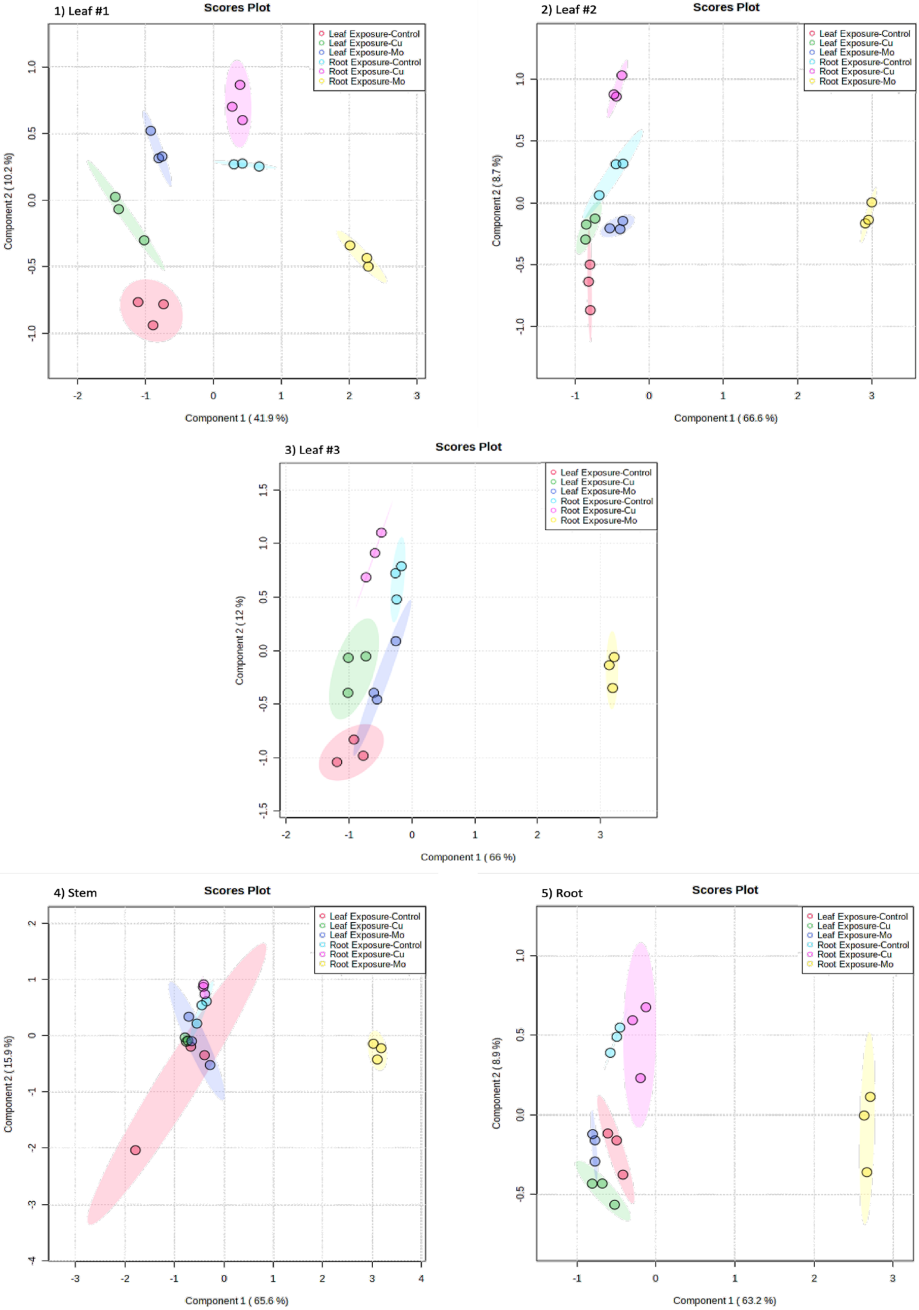
Partial least squares-discriminant analysis (PLS-DA) of metabolite concentrations in each plant tissue with different treatments and exposure routes.

**Figure 2:**
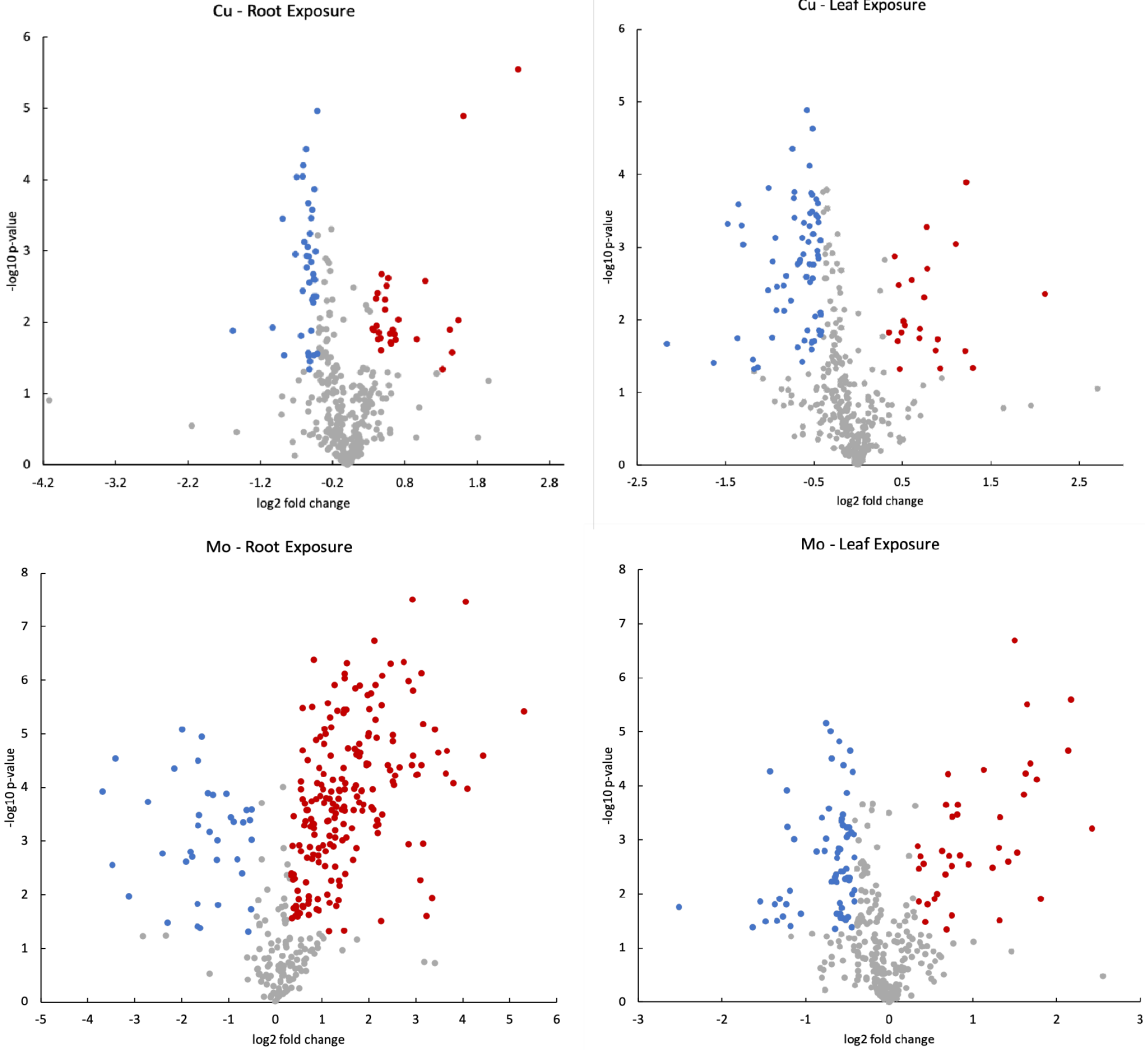
Volcano plots to visualize the relationship between significance (*p*-values < 0.05) and fold changes (FC) for each treatment. Gray points: not significant; red points: significant and FC ≥ 1.25; blue color points: FC ≤ 0.75.

A total of 82 metabolites, including 23 amino acids, 15 nucleobase/side/tides, 15 organic acids and phenolics, 13 sugar/sugar alcohol, 8 antioxidants, and 8 fatty acids in plants, were analyzed by LC–MS/MS for each tissue with different treatments. PLS-DA was employed to analyze the concentration of different metabolites in various tissues across six different treatment groups (Figure 1). Specifically, there was a noticeable and robust separation between the treatment involving Mo exposure through the root (represented by yellow dots) and all other treatments across all tissues analyzed. This separation aligns consistently with patterns observed in previous targeted proteomics study.^6^ The study revealed the distinction of Mo exposure through root treatment to wheat plant, as evidenced by depressed physiological measurements (yellowing and stunted growth), strong root uptake (more than 1000 μg/g Mo uptake) and root-to-leaf transport.^6^ The findings suggest that exposure to Mo via the root system significantly disrupted metabolic regulations, leading to a phenotypic response. Volcano plots were used to efficiently identify metabolites that display significant changes in expression levels alongside statistical significance across different treatments (Figure 2). In the volcano plot, data points were plotted based on their fold change (FC) on the *x*-axis and their significance level (*p*-values) on the *y*-axis. Gray spots indicate data points where the p-values were greater than 0.05, suggesting not statistically significant. Meanwhile, red and blue points denote significant upregulations (FC ≥ 1.25) and downregulations (FC ≤ 0.75), respectively. These red and blue points represent alterations in metabolomic expression that were both statistically significant and of biological relevance due to their substantial magnitude. The metabolites corresponding to these red and blue points were filtered as ″responsive metabolites″ for the respective treatments. Out of the 82 analyzed metabolites, 58 responsive metabolites were identified for Cu treatments and 71 responsive metabolites for Mo treatments.

**Figure 3:**
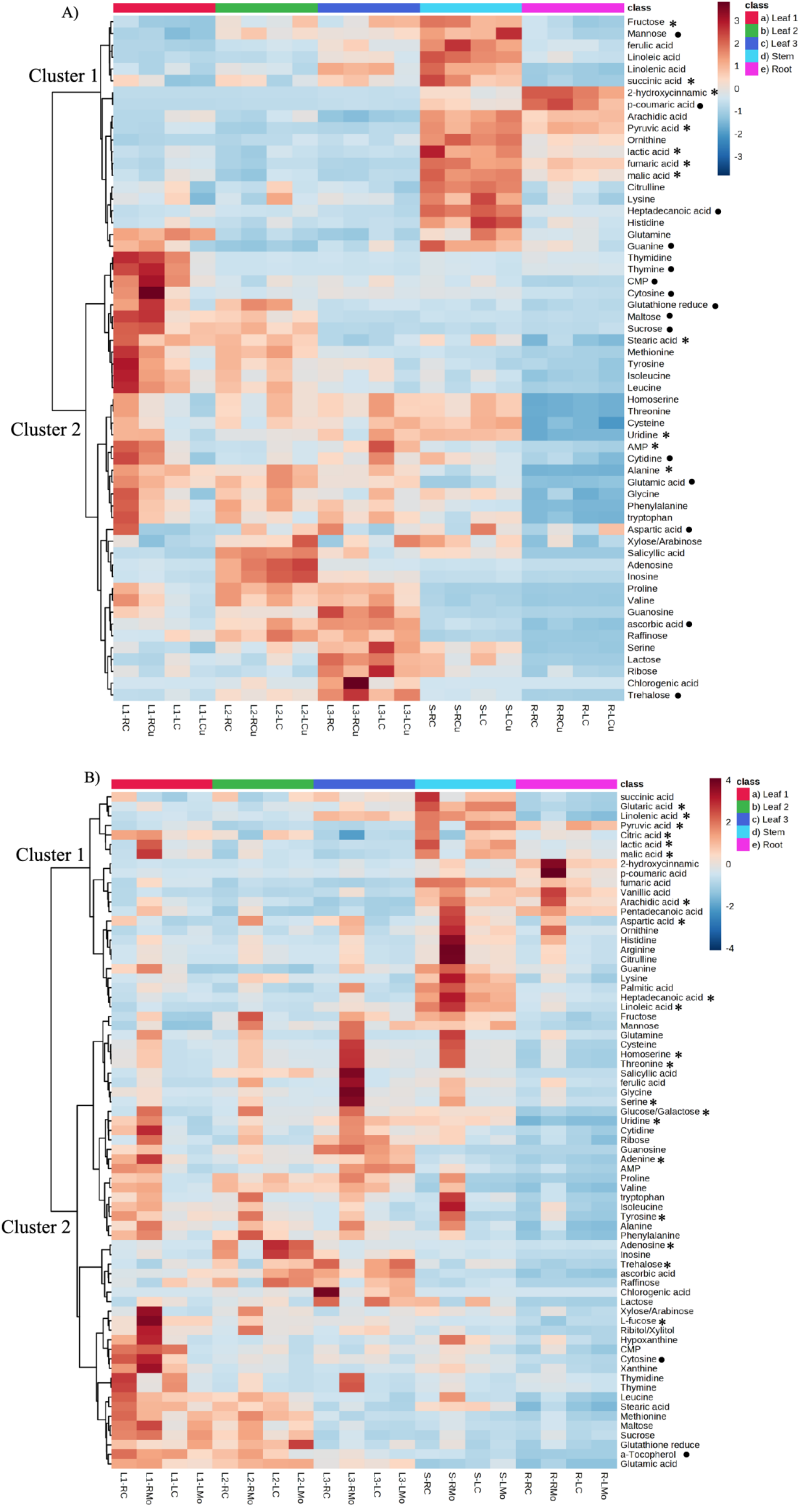
Heatmap of (A) 58 responsive metabolite concentrations in different plant tissues with Cu treatments. (B) 71 responsive metabolites concentrations in different plant tissues with Mo treatments. L1: leaf #1; L2: leaf #2; L3: leaf #3; S: stem; R: root; RC: root exposure control; LC: leaf exposure control; RCu: Cu exposure through root; LCu: Cu exposure through leaf; RMo: Mo exposure through root; LMo: Mo exposure through leaf. *: only responsive through root exposure; •: only responsive through leaf exposure.

Subsequently, a heatmap was generated to visualize the concentrations of these responsive metabolites across different tissues for Cu treatments (Figure 3A) and Mo treatments (Figure 3B). There were 58 responsive metabolites identified for Cu exposure, and 71 responsive metabolites were identified for Mo exposure. The heatmap depicts a tissue-specific distribution of responsive metabolites under both Cu and Mo treatments, suggesting distinct preferences for the accumulation of different metabolite groups in specific plant tissues. In general, Cluster 1, primarily composed of organic acids and fatty acids, indicates a tendency for the accumulation of these metabolites in stem or root tissues. Organic acids and fatty acids are commonly associated with energy storage and structural components in plant biology.^27,28^ The functions of these metabolites also aligns to the roles of stem and root tissues in energy storage and structural integrity within the plant’s overall physiology.^29^ On the other hand, Cluster 2, consisting mainly of sugars and amino acids, demonstrates a preference for accumulation in leaf tissues, particularly in L1 or L3. Sugars and amino acids play crucial roles in various processes vital to plant growth and development, particularly in photosynthesis and protein synthesis. Their abundance in leaves is associated with their essential functions within chloroplasts and the cytosol, which are particularly rich in leaf tissues.^30,31^

**Figure 4:**
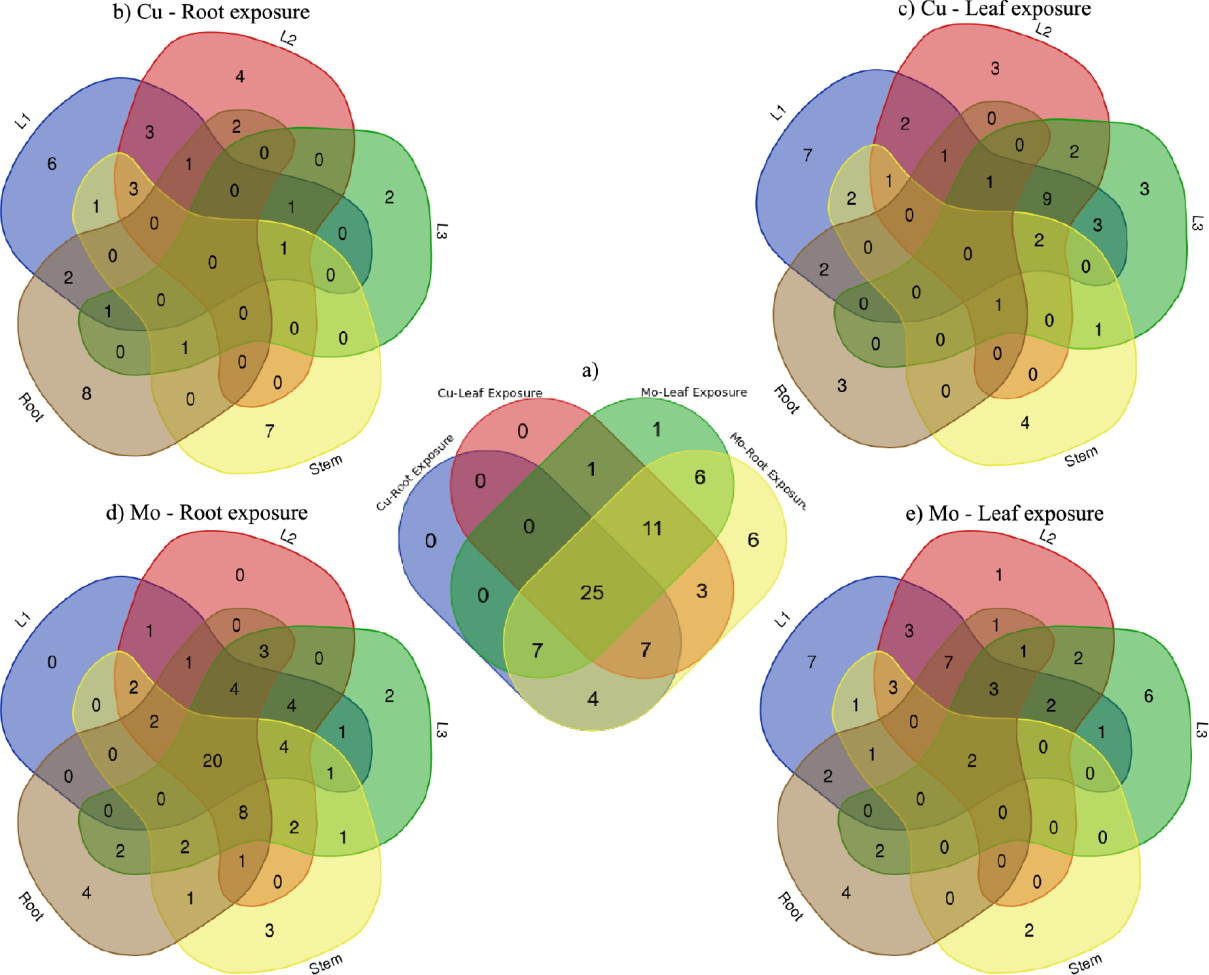
Venn diagram of (a) responsive metabolites with Cu and Mo exposure through root and leaf; (b) tissue-specific distribution of responsive metabolites with Cu exposure through root; (c) tissue-specific distribution of responsive metabolites with Cu exposure through leaf; (d) tissue-specific distribution of responsive metabolites with Mo exposure through root; and (e) tissue-specific distribution of responsive metabolites with Mo exposure through leaf.

The analysis using Venn diagrams showcased the overlaps and unique aspects of responsive metabolites among exposure to Cu and Mo, distinguishing between root and leaf exposures (Figure 4a). Notably, all 58 metabolites that showed responsiveness to Cu exposure were also identified as responsive metabolites in the Mo exposure groups. The overlap in responsive metabolites between the Cu and Mo exposure groups highlights a shared set of metabolic alterations induced by both Cu and Mo treatments, indicating potential similarities or interactions in their effects on the metabolic pathways. Among the 58 responsive metabolites for Cu exposure, 11 metabolites were responsive solely to root exposure, 15 metabolites were responsive solely to leaf exposure, and 32 metabolites were responsive to both root and leaf exposure. Among the 71 responsive metabolites for Mo exposure, 20 metabolites were responsive solely to root exposure, 2 metabolites (cytosine and a-tocopherol) were responsive solely to leaf exposure, and 49 metabolites were responsive to both root and leaf exposure. The exposure-specific responsive metabolites are labeled on heatmaps (Figure 3), with “*” as responsive exclusively due to root exposure while “•” as responsive exclusively due to leaf exposure. Metabolites without symbols were responsive to either root or leaf exposure. Notably, for Mo exposure, the higher number of metabolites specifically responding to root exposure signifies that this exposure route triggers a more active and distinct metabolic response in the plant compared to leaf exposure. This emphasizes the importance of considering the exposure route when assessing the effects of agrochemicals, particularly ENMs, on plant metabolomics, as different exposure approaches can lead to varying and distinctive metabolic responses. Another noteworthy observation is that among the 71 responsive metabolites identified across all treatments, a subset of 25 metabolites demonstrated responsiveness across all different treatments. This group of 25 metabolites comprises 15 amino acids, 4 sugars, 3 nucleobases/nucleosides/nucleotides, 2 phenols, and 1 antioxidant, highliting a core set of metabolites that consistently responded across various treatments.

To understand the tissue-specific response of these metabolites, Venn diagrams were also used to illustrate the responsive metabolites in each tissue for different treatments (Figure 4b–e). In the case of Mo exposure through roots (Figure 4d), among the responsive metabolites identified in different tissues, there were 20 metabolites that displayed responsiveness across every tissue analyzed, including 13 amino acids, 2 fatty acids, 2 antioxidants, 2 phenolics, and 1 organic acid. This uniform responsiveness in multiple tissues indicates a potentially systemic or global impact of Mo exposure on plant metabolism across various tissue types.

**Figure 5:**
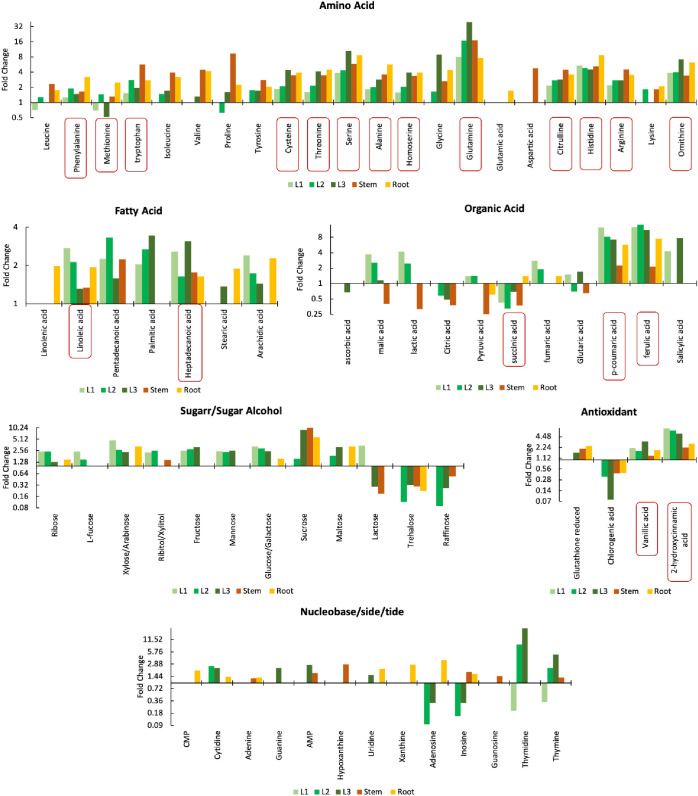
Fold change bar plots of 69 responsive metabolites (grouped by metabolite classes) in different plant tissues with Mo exposure through root. Metabolites highlighted with red squares are the ones responsive across all tissues.

To delve into the detailed regulation of responsive metabolites across various tissues for each treatment, fold change bar plots categorized by metabolite classes were generated (Figures 5, S3–S5). These bar plots illustrate the fold change, indicating significant alterations with a p-value below 0.05 and a fold change ≥1.25 or ≤0.75. Among various classes of metabolites, amino acids exhibited the most notable regulations, displaying significant fold changes and involvement across multiple tissues in response to different treatments. Mo exposure through roots resulted in a considerable upregulation (1.28 ≤ FC ≤ 39.60) of all analyzed amino acids across various plant tissues, except for leucine (in L1), methionine (in L1 and L3), and proline (in L2), which exhibited downregulation (0.52 ≤ FC ≤ 0.71) specifically in certain leaf samples under this exposure condition (Figure 5). Since Mo is actively involved in nitrogen metabolism, incorporated into molybdoenzymes to assimilate inorganic nitrogen into organic forms such as amino acids,^32^ Mo exposure through roots may enhance the activity of molybdoenzymes and induce the observed upregulation of amino acids. Moreover, the significant alterations in amino acid levels align with their crucial roles in the central metabolism of the plants. For example, glutamine, which showed the strongest upregulation (7.67 ≤ FC ≤ 39.60) in all tissues, serves as a nitrogen storage molecule and plays a vital role in nitrogen metabolism.^33^ Specifically, during stress conditions, glutamine acts as a vital nitrogen donor, providing readily available nitrogen for protein synthesis and other essential metabolic pathways, to cope with stress-induced changes by supporting crucial cellular processes under adverse conditions.^34^ This notable upregulation of glutamine reflects a Mo-induced stress due to the excess molybdenum uptake with root exposure, which aligns with the phytotoxic effect observed in our previous study.^6^ However, the amino acids in plants exposed to Mo via leaves were mostly downregulated (0.59 ≤ FC ≤ 0.75) across different tissues, except for methionine (in L1), cysteine (in L1), alanine (in stem), glutamine (in L1), glutamic acid (in stem), and ornithine (in L1, L2, and L3) that exhibited upregulation (1.28 ≤ FC ≤ 3.39) (Figure S5). These findings highlight contrasting patterns in amino acid regulation depending on exposure route.

In contrast to Mo exposure, Cu exposure through either root or leaf induced downregulation for most of the amino acids (Figures S3 and S4). However, ornithine, among all of the amino acids studied, stands out as the only one consistently upregulated across all tissues subjected to different Cu and Mo treatments. Ornithine plays a pivotal role in plant metabolism as it stands at the critical juncture of multiple essential metabolic pathways that lead to the production of various crucial compounds functional in several cellular processes related to growth, stress tolerance, and overall plant health.^35^ The noteworthy upregulation of ornithine despite the overall downregulation of other amino acids underscores its resilience mechanism, suggesting its involvement in stress adaptation and tolerance. This aligns with a study that indicated accumulation of ornithine delayed the stress- and age-dependent progression of leaf senescence by fueling the TCA cycle.^36^

### 3.2 Targeted Proteomics Analysis

**Figure 6:**
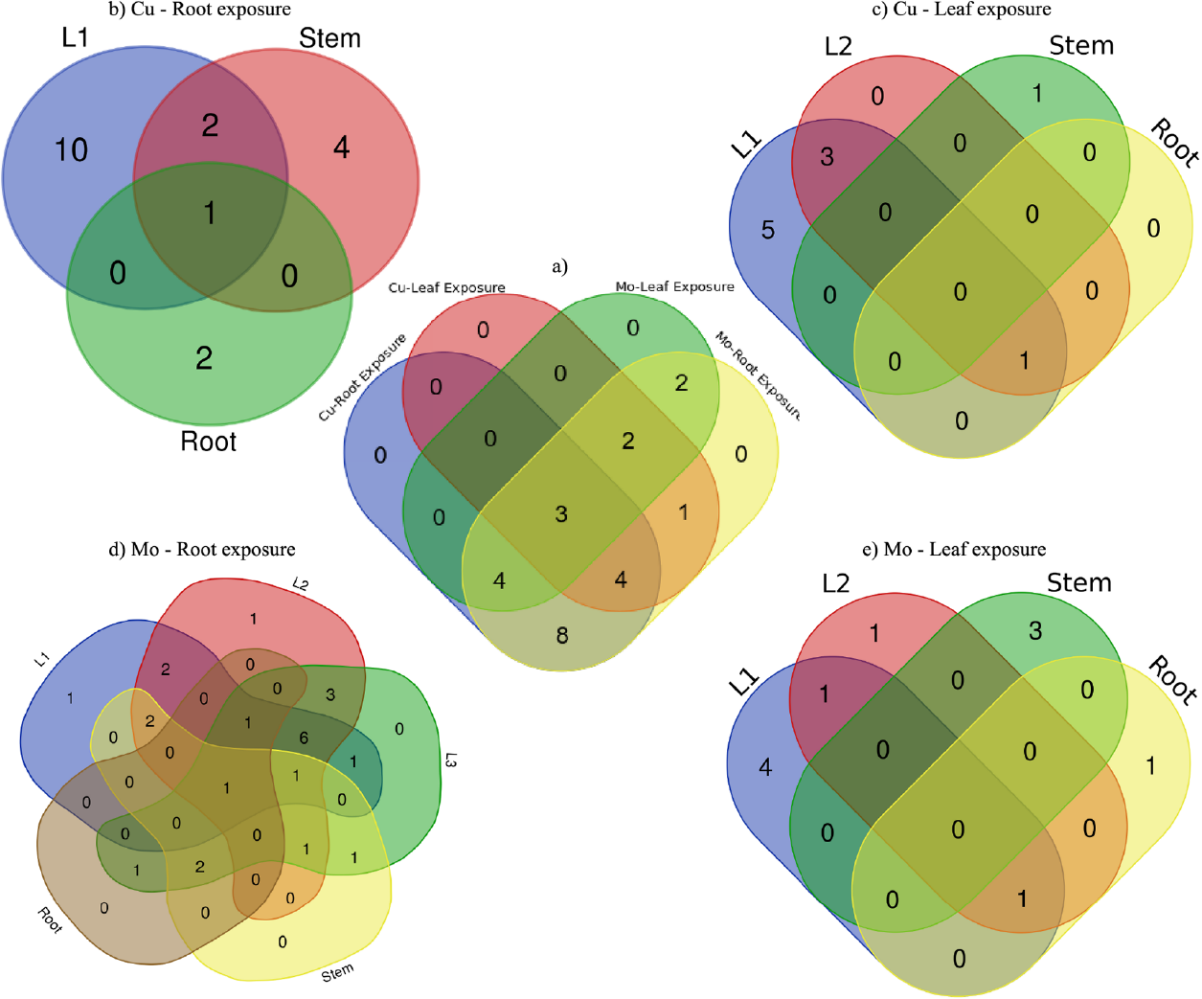
Venn diagram of (a) responsive proteins with Cu and Mo exposure through root and leaf; (b) tissue-specific distribution of responsive proteins with Cu exposure through root; (c) tissue-specific distribution of responsive proteins with Cu exposure through leaf; (d) tissue-specific distribution of responsive proteins with Mo exposure through root; and (e) tissue-specific distribution of responsive proteins with Mo exposure through leaf.

Similar to the identification of responsive metabolites, proteins meeting both criteria, significant changes in abundance with a p-value smaller than 0.05 and a fold change of ≥1.25 or ≤0.75, were identified as ″responsive proteins″. These proteins were considered to have undergone biologically relevant alterations in their concentrations in response to experimental treatments. The Venn diagram revealed distinct patterns in the responsiveness of proteins to different exposure treatments of Mo and Cu through root and leaf exposure methods (Figure 6a). For Mo treatments, all 24 proteins analyzed demonstrated responsiveness to Mo exposure through root application. In contrast, only 11 proteins showed responsiveness to Mo exposure through leaf application. This suggests a more limited impact or alteration in the abundance of proteins when Mo was applied through leaves compared with root exposure. For Cu treatments, 19 proteins were responsive when exposed through the root, while 10 proteins showed responsiveness when exposed through the leaf, with 7 proteins shared with root exposure. Notably, 3 proteins related to carbohydrate metabolism (P5-glycolysis cytosolic branch UGPase, P19-fructose-bisphosphate aldolase, and P20-Calvin cycle GAP) exhibited responsiveness across all treatments. The consistent response of these proteins across various treatments implied their significant role in maintaining carbohydrate metabolism under different environmental conditions or treatments. The regulation of these proteins may be a plant’s way of adapting its carbohydrate metabolism to optimize energy production, carbon fixation, or storage based on changing environmental cues or stressors.

Moreover, the tissue-specific distribution of responsive proteins reveals distinct patterns in their presence across different plant parts under Cu and Mo treatments through root and leaf exposure routes. For Cu exposure through roots, the responsive proteins predominantly appeared in L1 (13 proteins), followed by the stem (7 proteins), and fewer in the roots (3 proteins) (Figure 6b). On the other hand, Cu exposure through leaf showed a different distribution; 9 responsive proteins were observed in L1, with 4 shared proteins in L2, and one protein each in the stem and roots (Figure 6c). This indicates a stronger impact on protein abundance in the early emerged leaves compared to other tissues when Cu was applied, especially via the roots. Interestingly, under Mo treatments through root exposure, the responsive proteins were present in every tissue (Figure 6d). However, when Mo was applied through leaf exposure, the responsive proteins were absent in L3 (Figure 6e). The absence of responsive proteins in L3 (the last emerged leaf) for leaf exposure treatments with both Cu and Mo might be anticipated due to the shorter duration of exposure experienced by this leaf compared to that of the other tissues. The metabolic responses at the protein level might not have been fully induced or manifested within this shorter exposure time frame.

The detailed fold changes of responsive proteins in different treatments were visualized in bar plots, delineating tissue-specific responses (Figure S6). An observation similar to the metabolomics data emerged: Mo exposure through the root exhibited the most pronounced perturbations among the treatments, primarily characterized by upregulation trends, indicating an increased level of biosynthesis or accumulation of these proteins in response to the treatment. The aligned upregulation observed in both amino acids at the metabolomics level and proteins at the proteomics level indicates a significant interplay and interconnectedness between metabolomic and proteomic perturbations in the plant’s response to the treatments. For instance, in the case of Mo exposure through roots, the upregulation of specific amino acids provides the necessary building blocks for the increased synthesis of particular proteins. Simultaneously, the increased level of expression of these proteins enhances the assimilation of nitrogen, contributing to the elevated level of production of amino acids. This coordinated response indicates a potential bidirectional relationship, where changes in metabolite concentrations, such as amino acids, can influence or contribute to the modulation of protein expression levels, and conversely, alterations in protein expression can, in turn, impact the metabolic pathways involved, establishing a dynamic proteomic-to-metabolic-to-proteomic relationship.

### 3.3 Integrated Pathway Analysis

The joint pathway analysis using MetaboAnalyst 5.0, integrated with the KEGG pathway library, was conducted with the identified responsive metabolites and proteins. The analysis aimed to assess the impact of Cu and Mo treatments on metabolic pathways, considering both metabolomic and proteomic data. The threshold for impact value, determined through pathway topology analysis (Relative-betweenness Centrality), was established at 0.1, the cutoff point for identifying perturbed pathways based on their significance and relevance within the data set.^17^ Perturbed pathways resulting from the treatments are organized and are presented in Table S4 for Cu treatments and Table S5 for Mo treatments. These tables specified the perturbations observed in different tissues, offering a detailed breakdown of how these treatments influenced specific metabolic pathways across various plant tissues. The responsive metabolites and proteins involved in the perturbed pathways are also indicated in the tables, with root exposure exclusive (bold) or leaf exposure exclusive (underline) specified.

**Figure 7:**
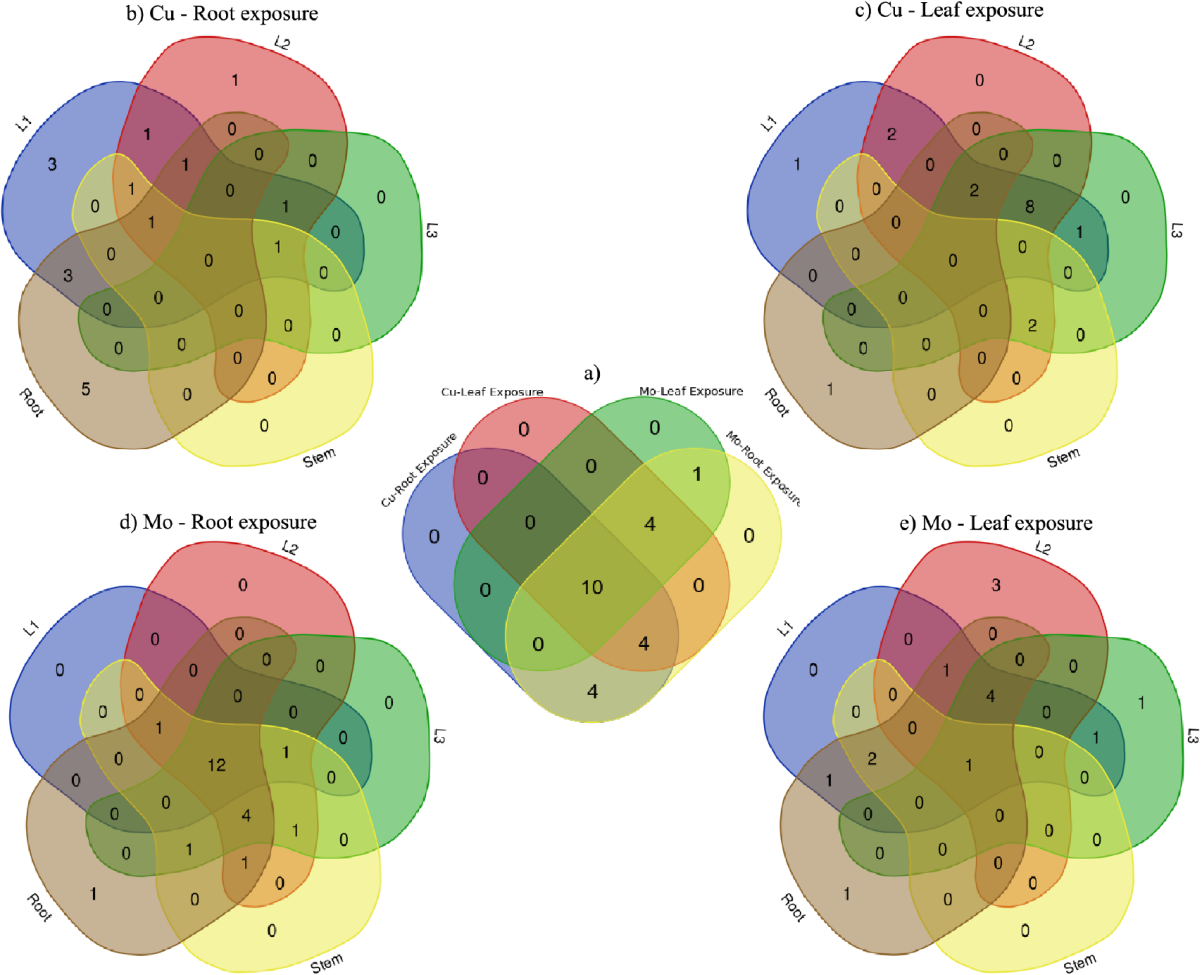
Venn diagrams of (a) perturbed pathways with Cu and Mo exposure through root and leaf; (b) tissue-specific distribution of perturbed pathways with Cu exposure through root; (c) tissue-specific distribution of responsive metabolites with Cu exposure through leaf; (d) tissue-specific distribution of perturbed pathways with Mo exposure through root; and (e) tissue-specific distribution of perturbed pathways with Mo exposure through leaf.

The analysis identified a total of 23 perturbed pathways across all treatments, categorized into 6 metabolic categories: amino acid metabolism (10 pathways), biosynthesis of secondary metabolites (4 pathways), carbohydrate metabolism (5 pathways), lipid metabolism (2 pathways), nucleotide metabolism (1 pathway), and translation (1 pathway) (Tables S4 and S5). Further insights from the Venn diagram (Figure 7) revealed differential and overlapping pathway perturbations for Mo and Cu exposure through root and leaf routes. For Mo treatments, exposure through roots involved perturbations across all 23 identified pathways while exposure through leaves affected 15 out of the 23 pathways. For Cu exposure, root and leaf exposure perturbed 22 of the 23 pathways, with 14 pathways shared and 4 pathways exclusively through either root or leaf exposure. Ten pathways were consistently perturbed across all four treatments: 8 related to amino acid metabolism (alanine, aspartate, and glutamate metabolism, arginine biosynthesis, tryptophan metabolism, cysteine and methionine metabolism, phenylalanine metabolism, glycine, serine, and threonine metabolism, arginine and proline metabolism, tyrosine metabolism), 1 associated with the biosynthesis of secondary metabolites (stilbenoid, diarylheptanoid, and gingerol biosynthesis), and 1 in carbohydrate metabolism (glyoxylate and dicarboxylate metabolism). Purine metabolism, a nucleotide metabolism pathway, was perturbed only by Mo exposure (both root and leaf routes). Additionally, 3 carbohydrate metabolism-related pathways (pyruvate metabolism, citrate cycle (TCA cycle), and glycolysis/gluconeogenesis) and 1 amino acid metabolism pathway (valine, leucine, and isoleucine biosynthesis) were perturbed only through root exposure, either with Cu or Mo. These findings highlight the complexity and specificity of the metabolic responses to different treatments. The shared perturbed pathways between Mo and Cu exposure methods suggest commonalities in their effects on metabolic pathways, while specific pathway perturbations indicate distinct impacts of each treatment method on the plant’s metabolic networks. In addition, tissue-specific analysis revealed a noteworthy observation regarding the perturbed pathways in response to Mo exposure through the roots (Figure 7d). For plants subjected to this treatment, 12 pathways showed perturbations consistently across all tissues, which were driven by the responsive metabolites identified. The consistent perturbations across various plant tissues indicate uniformity in the tissue-specific distribution of responsive metabolites under this treatment, which might serve as a driving factor behind the synchronized perturbations observed in those pathways.

**Figure 8:**
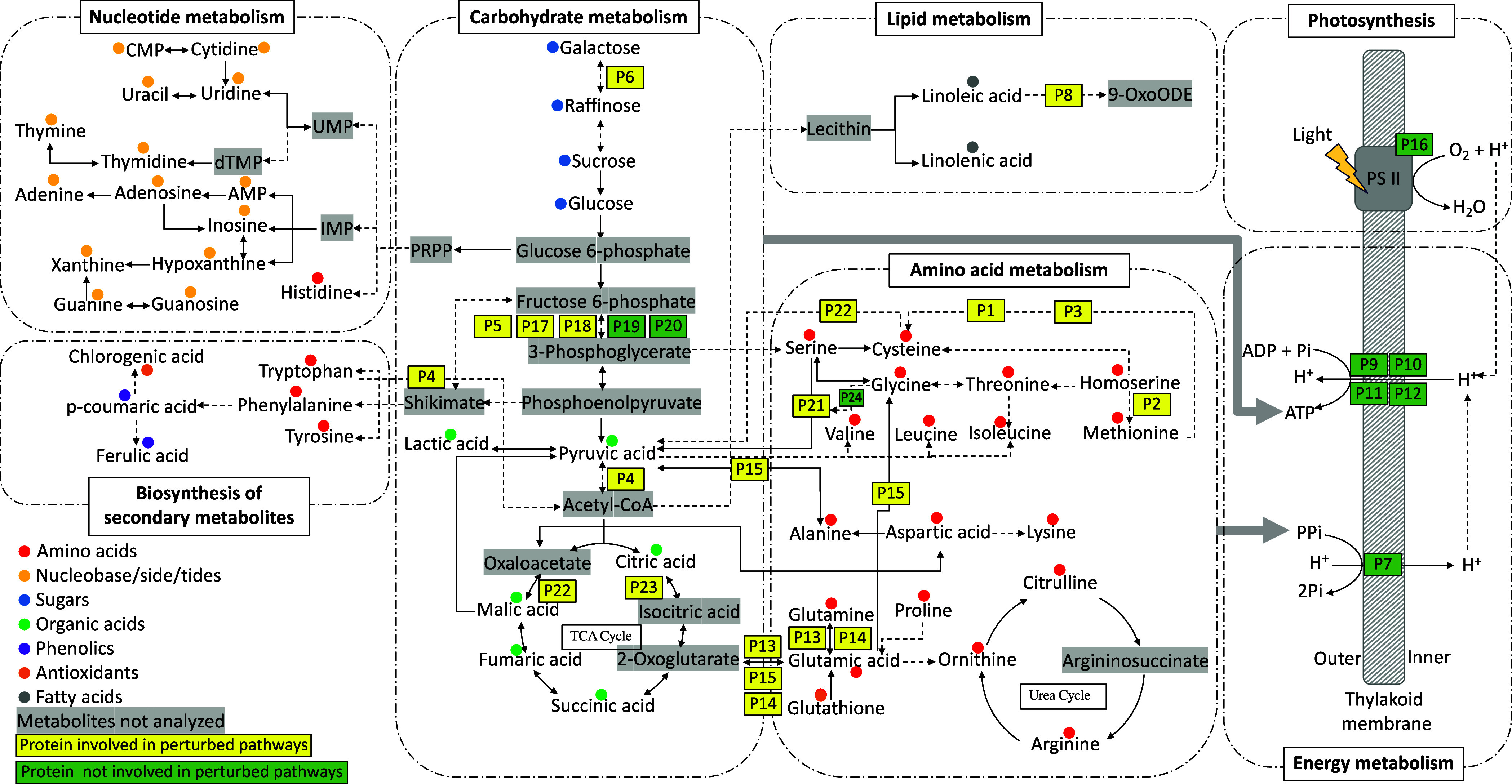
Pathway mapping of responsive metabolites and proteins based on KEGG.

Finally, pathway mapping was visualized based on KEGG pathway templates, indicating responsive metabolites and proteins on perturbed pathways across all treatments (Figure 8). The map integrates responsive metabolites and proteins to illustrate their involvement in various metabolic pathways and processes affected by the treatments. While some responsive proteins were not directly associated with the perturbed pathways identified through joint pathway analysis, they were labeled in green on the map, including 6 proteins actively involved in photosynthesis and energy metabolism. Amino acid metabolism-related pathways were most significantly perturbed, especially considering their involvement in various other crucial metabolic processes such as the TCA cycle and the electron transport chain. For example, amino acids can be converted into intermediates of the TCA cycle, such as pyruvate and oxaloacetate.^37^ This allows them to contribute to energy production through oxidative phosphorylation. The TCA cycle also provides intermediates for amino acid biosynthesis, demonstrating a two-way interaction between these pathways. In addition, through amino acid catabolism, NADH can donate electrons to the electron transport chain and ultimately generates ATP, the primary energy currency of the cell. In turn, the electron transport chain also plays a crucial role in maintaining cellular redox balance, which is essential for proper amino acid metabolism.^37^ This highlights the crucial role that amino acids play in maintaining overall cellular function and metabolism. In addition, the observed upregulation of responsive proteins primarily associated with amino acid metabolism could indeed offer an explanation for the active alterations in amino acid levels within the tissues. This interconnectedness between proteins and metabolites in amino acid metabolism highlights their intricate regulatory roles in shaping cellular metabolism and energy production, emphasizing their significance in the plant’s adaptive responses to different treatments.

### 3.4 Dose-Specific Regulation

Due to the reported yellowing and stunted growth caused by excess intake of Mo through root exposure at 6.25 mg/plant dose, an additional lower dose (0.6 mg/plant) that more closely represents field recommendation was added to our experiment for targeted metabolomics and proteomics analysis.^6^ The PLS-DA (Figure S7) indicated clear separations in metabolite concentrations within plant tissues exposed to high and low doses of Mo through root intake. This separation suggests distinct metabolic responses induced by different doses of Mo exposure. Although there was a significant overlap in responsive metabolites for Mo exposure through roots at high and low doses (66 responsive metabolites overlapped) (Figure S8B), the regulation patterns differed significantly between the two doses. For instance, at the low dose, compared to the control group where no Mo was introduced during plant growing, there was a prevalence of downregulation for amino acids (Figure S9A), especially isoleucine (0.03 ≤ FC ≤ 0.08), proline (0.04 ≤ FC ≤ 0.72), citrulline (0.27 ≤ FC ≤ 0.43), arginine (0.30 ≤ FC ≤ 0.47), and lysine (0.23 ≤ FC ≤ 0.52), in contrast to the upregulation observed at the high Mo dose. In addition, organic acids (low Mo dose: upregulation FC ≤ 27.91, downregulation FC ≥ 0.04; high Mo dose: upregulation FC ≤ 13.97, downregulation FC ≥ 0.25), antioxidants (low Mo dose: upregulation FC ≤ 17.33, downregulation FC ≥ 0.15; high Mo dose: upregulation FC ≤ 7.61, downregulation FC ≥ 0.08), and sugars (low Mo dose: upregulation FC ≤ 41.22, downregulation FC ≥ 0.34; high Mo dose: upregulation FC ≤ 10.20, downregulation FC ≥ 0.09) groups exhibited more pronounced regulations with the low dose (Figure S9A) compared to the high dose (Figure 5). These findings highlight the dose-dependent variations in the plant’s metabolic response to Mo exposure through the roots. Similar to responsive metabolites, the responsive proteins were mainly overlapped as well (23 out of 24), with the exception of aminotransferases peroxisomal (P 15) which was solely responsive to high dose. The overlapped responsive proteins induced differential regulation patterns with different dose. For example, glutamate dehydrogenase (P13) showcased downregulations (0.13 ≤ FC ≤ 0.54) with low dose while upregulations (1.38 ≤ FC ≤ 1.63) with high dose (Figure S9B). This provides a striking example of how different doses can elicit the opposite regulatory responses in this enzyme. This contrasting regulation of P13 likely leads to distinct changes in its catalytic activity and, subsequently, influences the conversion of glutamate.^38^ The magnitude of upregulation for glutamine (a product from glutamate) observed between high dose (7.67 ≤ FC ≤ 39.60) and low dose (1.50 ≤ FC ≤ 1.95) treatments could be attributed to these divergent expression patterns of glutamate dehydrogenase. These differences in pathway regulation provide a potential explanation for the varied growth response of plants subjected to different doses of Mo. Dose-specific effects of copper were not investigated in this study due to the absence of observed phenotypic toxicity at the original dose chosen for both copper and molybdenum. However, exploring multiple doses, including a higher dose that could potentially induce toxicity in plants, could have elucidated dose-dependent effects of Cu, thereby enhancing our understanding of copper’s dual role as a nanopesticide and nutrient.

The multiomics investigation into the effects of Mo- and Cu-based ENMs exposure on plant metabolomics and proteomics with targeted analysis approaches presented a multilayered understanding of the intricate responses within different tissues, doses, and exposure routes. The joint pathway analysis unveiled 23 perturbed pathways across all treatments. Notably, Mo exposure through roots impacted all identified pathways with 12 pathways consistently perturbed across all tissues. In contrast, Mo exposure through leaves influenced 15 pathways, with only one pathway shared across all tissues. This underscores the significant influence of the exposure route and highlights the tissue-specific inducement of metabolic and proteomic responses in the plant’s reaction. In addition, pathway mapping visualized the involvement of responsive metabolites and proteins in perturbed pathways across all treatments, emphasizing the significance of amino acid metabolism. The observed upregulation of proteins associated with amino acid metabolism explained alterations in amino acid levels, highlighted a dynamic proteomic-to-metabolic-to-proteomic relationship, and suggested an intricate interplay between metabolomic and proteomic responses. Metabolites also showcased distinct tissue preferences, with organic acids and fatty acids being more prevalent in stem or root tissues, while sugars and amino acids were abundant in leaves, emphasizing their roles in energy storage, structural integrity, photosynthesis, and protein synthesis. Notably, the contrasting expression changes of key enzymes, exemplified by the case of glutamate dehydrogenase (P13), between different doses of Mo through root exposure highlighted dose-dependent regulatory patterns in enzymes and metabolites.

In summary, this extensive multiomics analysis provides invaluable insights into the intricate and interconnected mechanisms governing plant responses to Mo- and Cu-based ENMs exposure. The tissue specificity, exposure methods and dose dependencies, and pathway perturbations uncovered here contribute significantly to understanding plant metabolism under various stress conditions, offering crucial guidance for agricultural practices, environmental safety, and further research on the impact of nanomaterials on plants.

## Supporting Information Available

Detailed information on sample preparation and LC–MS/MS analytical methods for targeted metabolomics with the chromatograph of each group of metabolites; fold change bar plot of responsive metabolites for Cu exposure through root or leaf and Mo exposure through leaf; fold change bar plots of 24 responsive proteins in different plant tissues with Cu and Mo exposure through root or leaf routes; data analysis of targeted metabolomics and targeted proteomics of plants exposed to low-dose Mo ENMs treatments; and results of joint-pathway analysis for various treatments, elucidating the interconnected pathways affected by Cu and Mo exposures (PDF)
