# Supplementary material for: Integrating Targeted Metabolomics and Targeted Proteomics to Study the Responses of Wheat Plants to Engineered Nanomaterials
Source: ACS Agric Sci Technol. 2024 Apr 2;4(4):507–20. doi: 10.1021/acsagscitech.4c00046 (PMC11022172; doi:10.1021/acsagscitech.4c00046)
Supplement: Supplementary file 1 — as4c00046_si_001.pdf [file as4c00046_si_001.pdf]

Supporting Information for

Integrating Targeted Metabolomics and Targeted Proteomics to Study the Responses of Wheat Plants to Engineered Nanomaterials

Weiwei Li and Arturo A. Keller*

Bren School of Environmental Science and Management, University of California at Santa Barbara, Santa Barbara, California 93106, USA

*Corresponding author: Tel: +1 805 893 7548; Fax: +1 805 893 7612. Email address: arturokeller@ucsb.edu

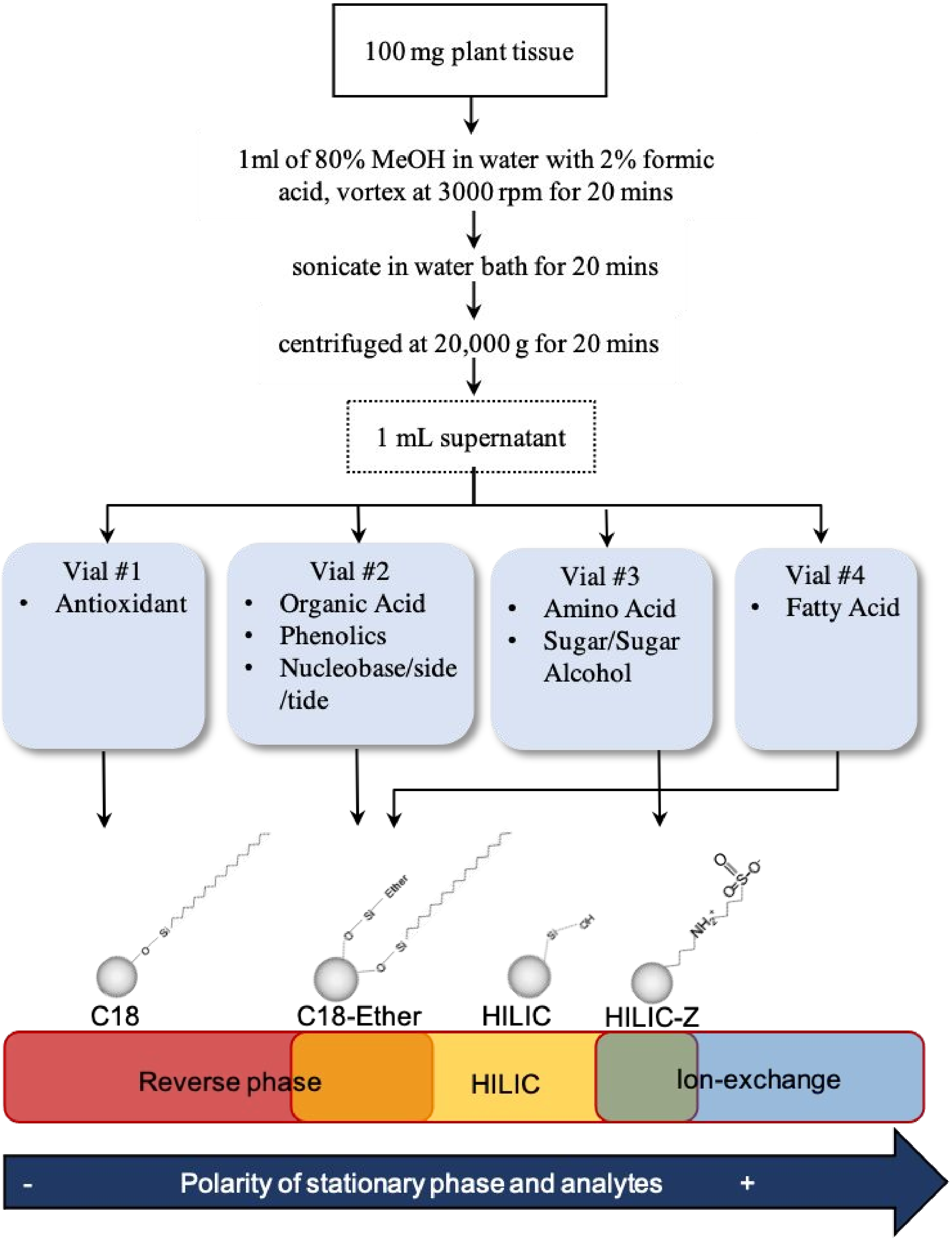

Figure S1. Flowchart of sample preparation for targeted metabolomics analysis.

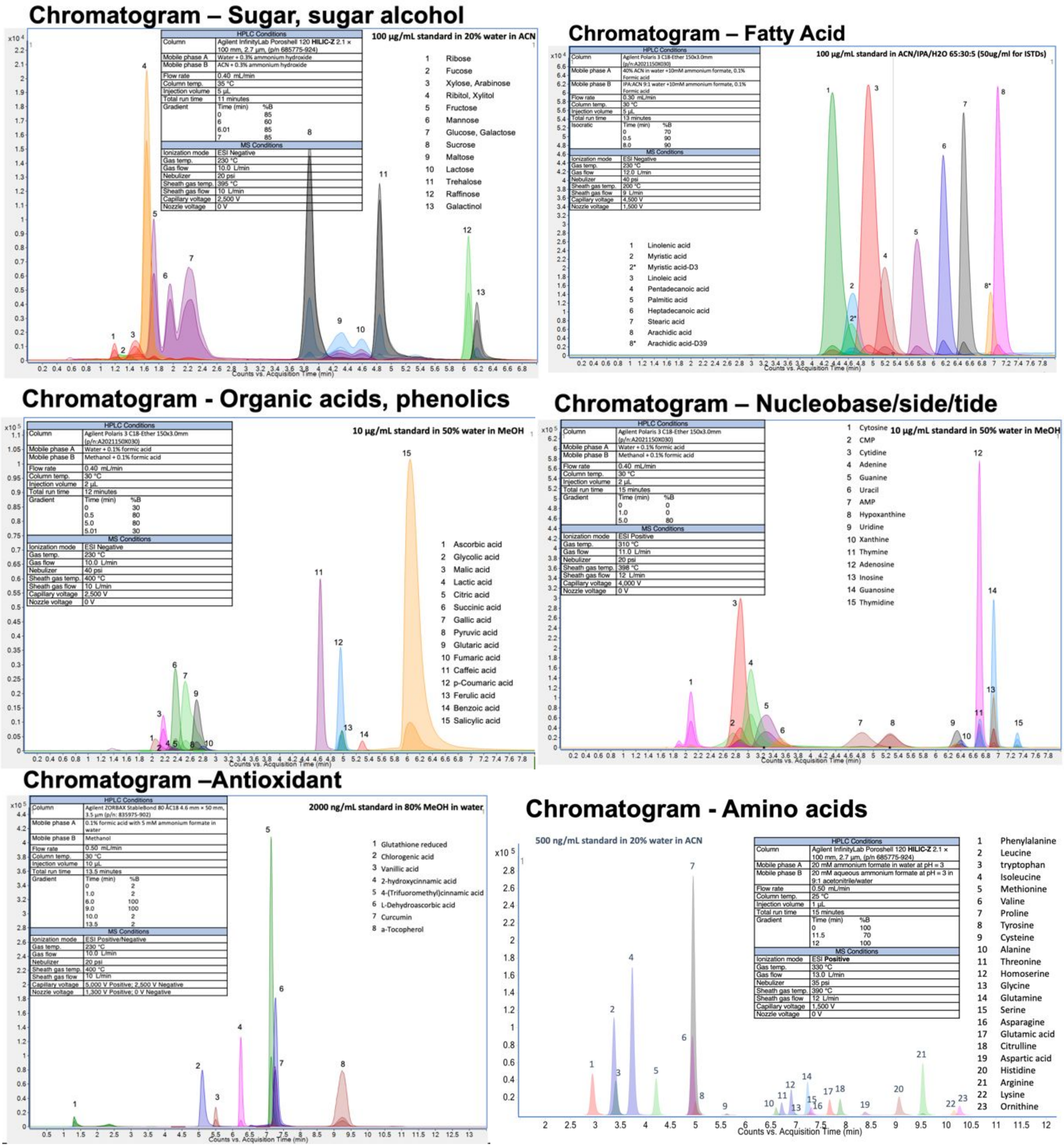

Figure S2. LC-MS/MS chromatograph of 6 groups of metabolites.

Table S1. Metabolite analytes grouped in vials with reconstitution solvent and LCMS column/mobile phase information.

Vial #	Analytes	Sample solvent	LCMS column	Mobile phase A	Mobile phase B	LOD	MDL
1	**Antioxidants**	80% MeOH in water	Agilent ZORBAX StableBond 80 ÅC18 4.6 mm × 50 mm, 3.5 μm	0.1% formic acid with 5 mM ammonium formate in water	Methanol	**(ng/mL)**	**(ng/g)**
	Glutathione reduced					0.05	0.5
	Chlorogenic acid					0.005	0.05
	Vanillic acid					0.6	6
	2-hydroxycinnamic acid					0.005	0.05
	4-(Trifuoromethyl)cinnamic acid					0.001	0.01
	L-Dehydroascorbic acid					0.01	0.1
	Curcumin					0.004	0.04
	a-Tocopherol					500	5000
2	**Organic Acid**	50% MeOH in water	Agilent Polaris 3 C18-Ether 150x3.0mm	0.1% formic acid in water	0.1% formic acid in MeOH	**(ug/mL)**	**(ug/g)**
	glycolic acid					4.60	46.02
	malic acid					0.52	5.16
	Citric acid					3.39	33.94
	lactic acid					6.39	63.92
	succinic acid					0.38	3.82
	Pyruvic acid					6.79	67.85
	Glutaric acid					2.85	28.46
	fumaric acid					0.35	3.52
	ascorbic acid					6.79	67.93
	Caffeic acid					0.58	5.79
	ferulic acid					0.34	3.40
	benzoic acid					0.81	8.10
2	**Phenolics**	50% MeOH in water	Agilent Polaris 3 C18-Ether 150x3.0mm	0.1% formic acid in water	0.1% formic acid in MeOH	**(ug/mL)**	**(ug/g)**
	Gallic acid					1.61	16.10
	p-Coumaric acid					0.342	3.42
	Salicylic acid					0.346	3.46
2	**Nucleobase/side/tide**	50% MeOH in water	Agilent Polaris 3 C18-Ether 150x3.0mm	0.1% formic acid in water	0.1% formic acid in MeOH	**(ug/mL)**	**(ug/g)**
	Cytosine					0.87	8.72
	CMP					0.32	3.22
	Cytidine					2.90	29.00
	Adenine					0.70	6.98
	Guanine					0.64	6.43
	uracil					0.66	6.56
	AMP					0.33	3.26
	Hypoxanthine					0.23	2.27
	Uridine					0.90	8.99

	Xanthine					0.26	2.62
	Adenosine					2.50	25.04
	Thymine					1.55	15.50
	Guanosine					2.79	27.92
	Inosine					0.58	5.78
	Thymidine					0.41	4.10
3	**Amino Acids**	80% ACN in water	Agilent InfinityLab Poroshell 120 HILIC-Z 2.1 × 100 mm, 2.7 μm	water +10% stock (Stock: 200 mM ammonium formate in water with formic acids adjusted pH = 3	ACN+ 10% stock	**(ng/mL)**	**(ng/g)**
	Phenylalanine					0.50	5.00
	Leucine					0.01	0.05
	tryptophan					0.01	0.05
	Isoleucine					0.01	0.05
	Methionine					0.05	0.50
	Valine					0.40	4.00
	Proline					1.00	10.00
	Tyrosine					0.15	1.50
	Cysteine					5.00	50.00
	Alanine					0.01	0.05
	Threonine					0.40	4.00
	Homoserine					0.15	1.50
	Glycine					15.00	150.00
	Glutamine					0.50	5.00
	Serine					10.00	100.00
	Asparagine					1.00	10.00
	Glutamic acid					0.01	0.05
	Citrulline					0.15	1.50
	Aspartic acid					0.01	0.05
	Histidine					0.25	2.50
	Arginine					0.01	0.10
	Lysine					0.01	0.05
	Ornithine					0.01	0.10
3	**Sugar/Sugar Alcohols**	80% ACN in water	Agilent InfinityLab Poroshell 120 HILIC-Z 2.1 × 100 mm, 2.7 μm	0.3% ammonium hydroxide in water	0.3% ammonium hydroxide in ACN	**(ug/mL)**	**(ug/g)**
	Ribose					3.95	39.51
	Fucose					3.91	39.10
	Xylose, Arabinose					3.20	32.01
	Ribitol, Xylitol					0.65	6.46
	Fructose					2.60	25.95
	Mannose					2.05	20.46
	Glucose, Galactose					3.07	30.72
	Sucrose					1.08	10.76
	Maltose					2.23	22.27
	Lactose					22.94	229.42

	Trehalose					0.65	6.47
	Raffinose					1.26	12.56
	Galactinol					4.36	43.60
4	**Fatty Acid**					**(ug/mL)**	**(ug/g)**
	Linolenic acid	ACN/IPA/H2O =65:30:5	Agilent Polaris 3 C18-Ether 150x3.0mm	40% ACN in water +10mM ammonium formate, 0.1% Formic acid	IPA:ACN = 9:1 +10mM aqueous ammonium fo rmate, 0.1% Formic acid	3.35	33.51
	myristic acid					3.74	37.40
	Linoleic acid					2.45	24.48
	Pentadecanoic acid					2.66	26.63
	Palmitic acid					13.63	136.33
	Heptadecanoic acid					10.91	109.07
	Stearic acid					12.91	129.10
	Arachidic acid					7.86	78.56

Table S2. The LC-MS/MS analysis parameters for metabolomics analysis

Compound	Retention time (min)	Precursor ion (m/z)	Product ions				Fragmentor (V)
			Quant ion (m/z)	Collision energy (V)	Qual ion (m/z)	Collision energy (V)	
**Antioxidants**							
Glutathione reduced	1.31	308.1	179	12	162	16	91
Chlorogenic acid	5.10	353.1	191.1	16	-	-	102
Vanillic acid	5.50	367.1	217.1	8	149.1	16	112
2-hydroxycinnamic acid	6.24	167	152.1	12	108	20	82
4-(Trifuoromethyl)cinnamic acid	7.14	163	119.1	12	117.1	28	81
L-Dehydroascorbic acid	7.25	173	158.1	12	-	-	174
Curcumin	7.27	215	171.1	12	151.1	20	87
a-Tocopherol	9.24	431.4	165.1	24	69.1	40	142
**Organic Acids/Phenolics**							
glycolic acid	2.04	75	47	8	72.9	8	46
malic acid	2.07	133	114.9	8	71	16	76
Citric acid	2.17	191	110.8	12	86.9	16	82
lactic acid	2.23	89.1	43.1	4	-	-	66
succinic acid	2.31	117	72.9	12	98.9	8	66
Pyruvic acid	2.36	87	43.1	4	-	-	66
Gallic acid	2.49	169	125.1	12	79	24	92
Glutaric acid	2.62	131	86.9	12	112.9	8	71
fumaric acid	2.67	115	70.9	4	-	-	56
ascorbic acid	2.67	175	114.9	12	-	-	87

Caffeic acid	4.58	179	135.1	16	-	-	94
p-coumaric acid	4.87	163	119.1	16	93.1	36	87
ferulic acid	5.09	193.1	134.1	16	178.1	12	87
benzoic acid	5.21	121	77.1	12	-	-	77
Salicyllic acid	5.96	137	93	20	65.1	36	82
**Nucleobase/side/tide**							
Cytosine	1.94	112.1	95	20	40.1	20	84
CMP	2.76	324.1	112	16	95	40	84
Cytidine	2.90	244.1	112	12	95	40	84
Adenine	3.08	136.1	119	24	92	32	84
Guanine	3.34	152.1	135	20	110	24	84
uracil	3.52	113	70	10	96	20	84
AMP	4.84	348.1	136	20	97	32	84
Hypoxanthine	5.28	137	110	24	55.1	36	148
Uridine	6.33	245.1	113	8	70	40	84
Xanthine	6.40	153	110	20	55.1	36	84
Adenosine	6.67	268.1	136	20	119	40	84
Thymine	6.71	127.1	110	16	54.1	28	84
Guanosine	6.91	284.1	152	12	135	40	84
Inosine	6.91	269.1	137	16	110	40	84
Thymidine	7.28	243.1	127	8	117	8	84
**Amino acids**							
Phenylalanine	2.95	166.1	120.1	13	103	29	80
Leucine	3.38	132.1	86.1	9	30.2	17	75
Tryptophan	3.41	205.1	188	8	146	20	80
Isoleucine	3.75	132.1	86.1	9	44.2	25	75
Methionine	4.22	150.1	104	9	56.1	17	75
Valine	4.95	118.1	72.1	9	55.1	25	70
Proline	4.96	116.1	70.1	17	43.2	37	75
Tyrosine	5.01	182.1	136.1	13	91.1	33	85
Cysteine	5.63	122	59.1	29	76	13	65
Alanine	6.61	90.1	44.2	9	45.3	40	40
Threonine	6.72	120.1	74.1	9	56.1	17	75
Homoserine	6.91	120.1	74.1	9	56.1	21	70
Glycine	7.00	76	30.3	12	-	-	35
Glutamine	7.23	147.1	84.1	17	130.1	9	80
Serine	7.26	106.1	88.1	8	42.2	24	67
Asparagine	7.31	133.1	87.1	5	74	17	75

Glutamic acid	7.68	148.1	84.1	17	130	5	75
Citrulline	7.89	176.1	159.1	9	70.1	25	80
Aspartic acid	8.38	134	88.1	9	74	13	70
Histidine	9.06	156.1	110.1	13	83.1	29	90
Arginine	9.54	175.1	70.1	24	60.1	12	100
Lysine	10.16	147.1	84.1	17	130.1	9	75
Ornithine	10.28	133.1	116	8	70	20	76
**Sugar and Sugar Alcohol**							
Ribose	1.18	149	89	4	-	-	76
L-fucose	1.35	163.1	89	0	59.1	12	76
Xylose/Arabinose*	1.43	149	89	4	-	-	76
Ribitol/Xylitol*	1.61	151.1	89	8	71.1	16	97
Fructose	1.72	179.1	89	4	-	-	71
Mannose	1.93	179.1	89	16	-	-	71
Glucose/Galactose*	2.19	179.1	89	16	-	-	71
Sucrose	3.81	341.1	179	20	-	-	148
Maltose	4.26	341.1	161.1	4	-	-	123
Lactose	4.57	341.1	161.1	4	-	-	123
Trehalose	4.79	341.1	179	12	-	-	154
Raffinose	6.03	503.2	179	20	221	32	174
Galactinol	6.17	341.1	179	12	-	-	133
**Fatty Acids**							
Linolenic acid	4.33	323.2	277.1	4	45.1	40	87
myristic acid	4.64	273.2	227.2	4	45.1	8	56
Linoleic acid	4.91	325.2	279.1	4	45.1	28	87
Pentadecanoic acid	5.17	287.2	241.2	4	45.1	16	71
Palmitic acid	5.70	301.2	255.2	4	45.1	20	36
Heptadecanoic acid	6.14	315.3	269.2	4	45.2	28	76
Stearic acid	6.49	329.3	283.2	4	45.1	32	72
Arachidic acid	7.05	357.3	311.3	4	45.1	32	82

Table S3. List of selected 24 targeted proteins with related pathways

Pathway ID	Pathway	Protein ID	Protein	Accession Number
A	Amino acid metabolism	P1	AA degradation methionine	AT3G23810
		P2	AA synthesis methionine	AT5G17920
		P3	S-adenosylmethionine synthase	AT1G02500
B	Fermentation	P4	aldehyde dehydrogenase	AT1G23800

C	Glycolysis	P5	glycolysis cytosolic branch UGPase	AT2G36460
		P6	glycolysis cytosolic branch aldolase	AT5G17310
D	H+ transporting pyrophosphatase	P7	transport H+ transporting pyrophosphatase	AT1G15690
E	Hormone metabolism	P8	lipoxygenase	AT1G55020
F	Mitochondrial electron transport / ATP synthesis	P9	transport p- and v-ATPase	AT1G78900
		P10	ATP synthase delta chain	AT4G09650
		P11	ATP synthase beta subunit	AT5G08670
		P12	ATP synthase F1-ATPase	AT2G07698
G	Nitrogen-metabolism	P13	glutamate dehydrogenase	AT5G07440
		P14	glutamate synthase ferredoxin dependent	AT5G04140
H	Photorespiratory pathway	P15	aminotransferases peroxisomal	AT1G70580
		P16	photosystem II stability/assembly factor	AT5G23120
I	Photosynthesis / Calvin Cycle	P17	calvin cycle aldolase	AT2G21330
		P18	calvin cycle FBPase	AT3G54050
		P19	fructose-bisphosphate aldolase	AT2G36460
		P20	calvin cycle GAP	AT3G26650
J	Redox	P21	catalase	AT1G20620
K	TCA / org transformation	P22	malate dehydrogenase	AT5G43330
		P23	TCA aconitase	AT4G35830
L	Tetrapyrrole biosynthesis	P24	tetrapyrrole synthesis prophobilinogen	AT5G08280

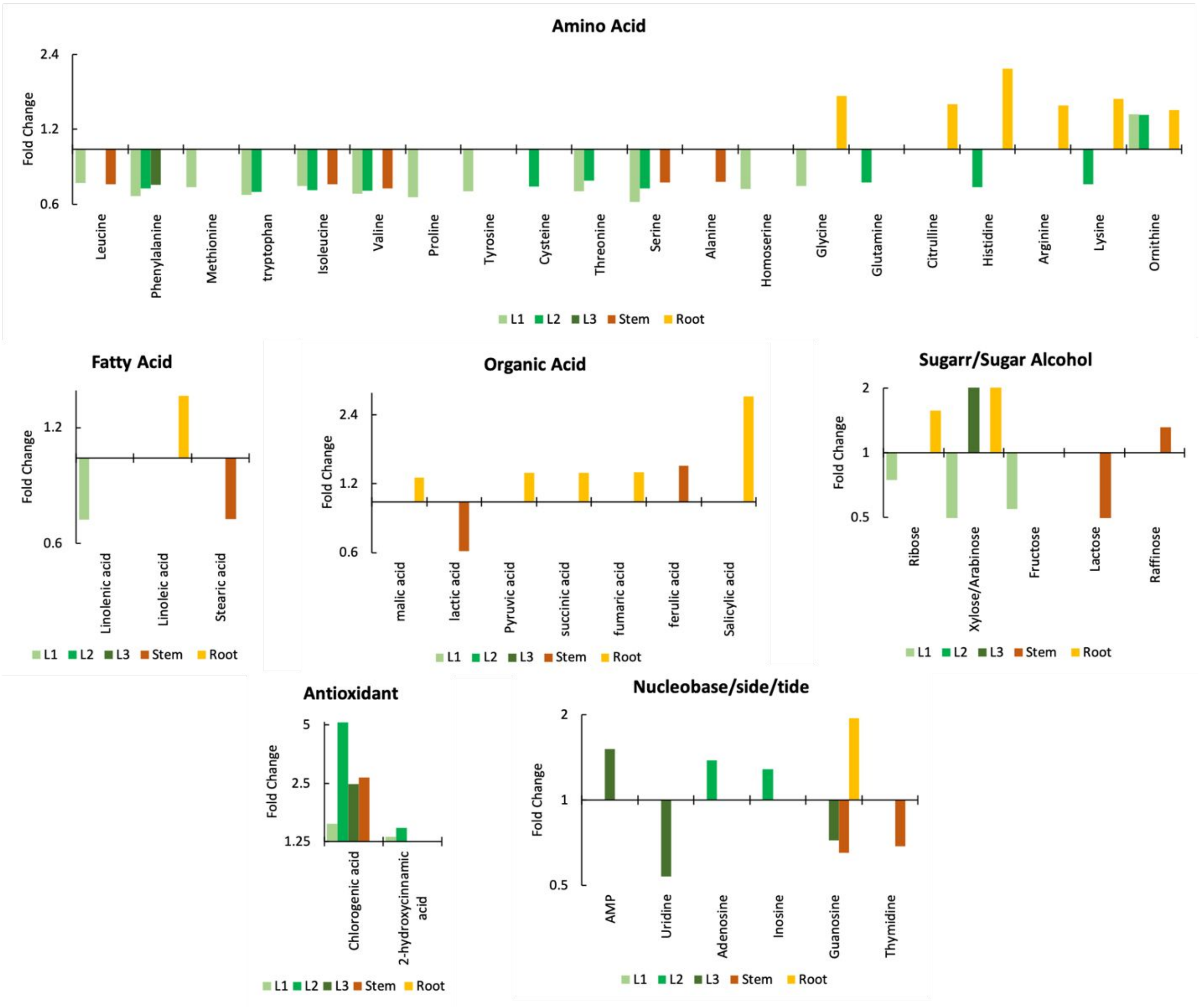

Figure S3. Fold change bar plots of 43 responsive metabolites in different plant tissues with Cu exposure through root.

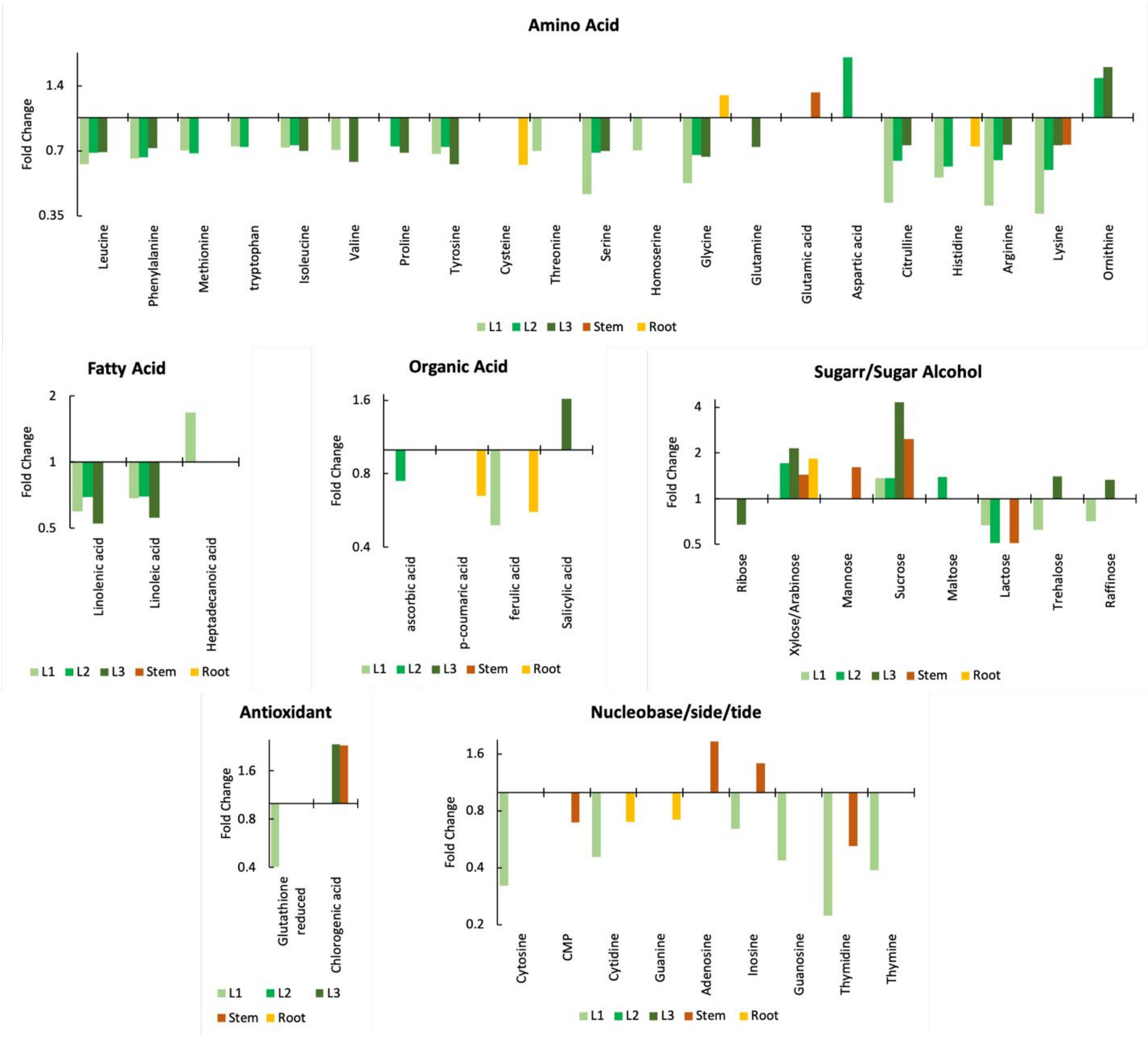

Figure S4. Fold change bar plots of 47 responsive metabolites in different plant tissues with Cu exposure through leaf.

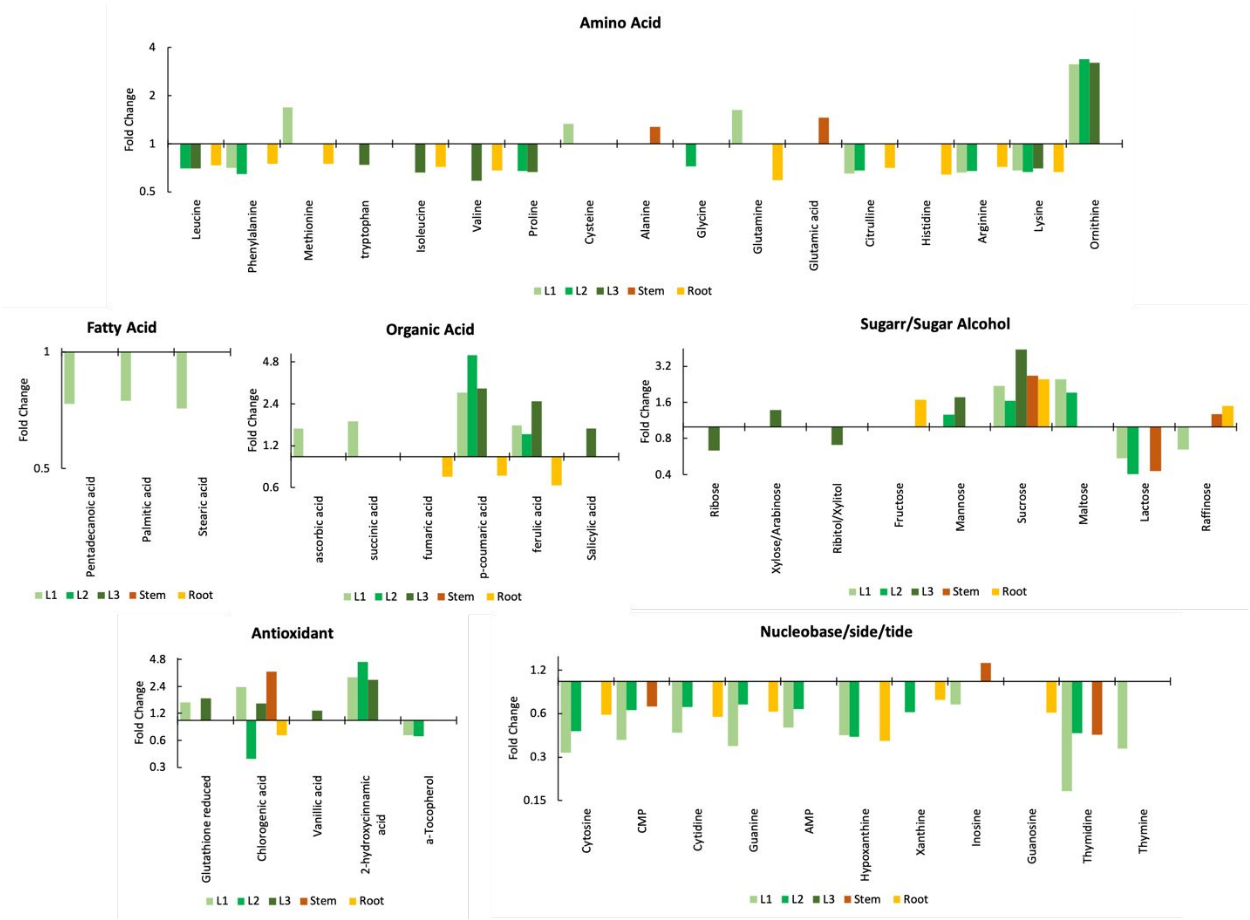

Figure S5. Fold change bar plots of 51 responsive metabolites in different plant tissues with Mo exposure through leaf.

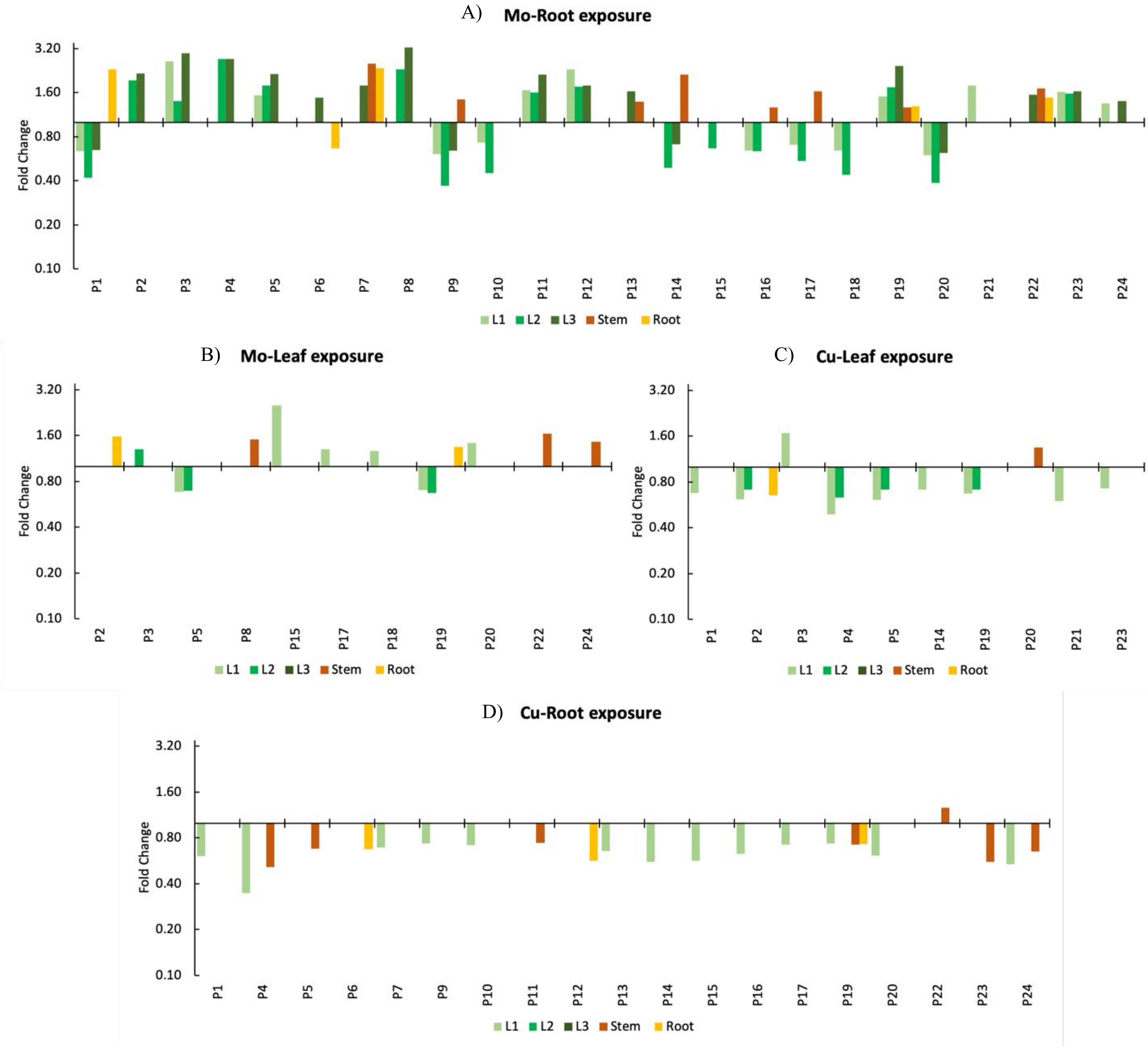

Figure S6. Fold change bar plots of A) 24 responsive proteins in different plant tissues with Mo exposure (6.25 mg/plant) through Root; B) 11 responsive proteins in different plant tissues with Mo exposure through leaf; C) 10 responsive proteins in different plant tissues with Cu exposure through leaf; and D) 19 responsive proteins in different plant tissues with Cu exposure through root. P1: AA degradation methionine; P2: AA synthesis methionine; P3: S-adenosylmethionine synthase; P4: aldehyde dehydrogenase; P5: glycolysis cytosolic branch UGPase; P6: glycolysis cytosolic branch aldolase; P7: transport H+ transporting pyrophosphatase; P8: lipoxygenase; P9: transport p- and v-ATPase; P10: ATP synthase delta chain; P11: ATP synthase beta subunit;

P12: ATP synthase F1-ATPase; P13: glutamate dehydrogenase; P14: glutamate synthase ferredoxin dependent; P15: aminotransferases peroxisomal; P16: photosystem II stability/assembly factor HCF136; P17: calvin cycle aldolase; P18: calvin cycle FBPase; P19: fructose-bisphosphate aldolase; P20: calvin cycle GAP; P21: catalase; P22: malate dehydrogenase; P23: TCA aconitase; P24: tetrapyrrole synthesis prophobilinogen deaminase.

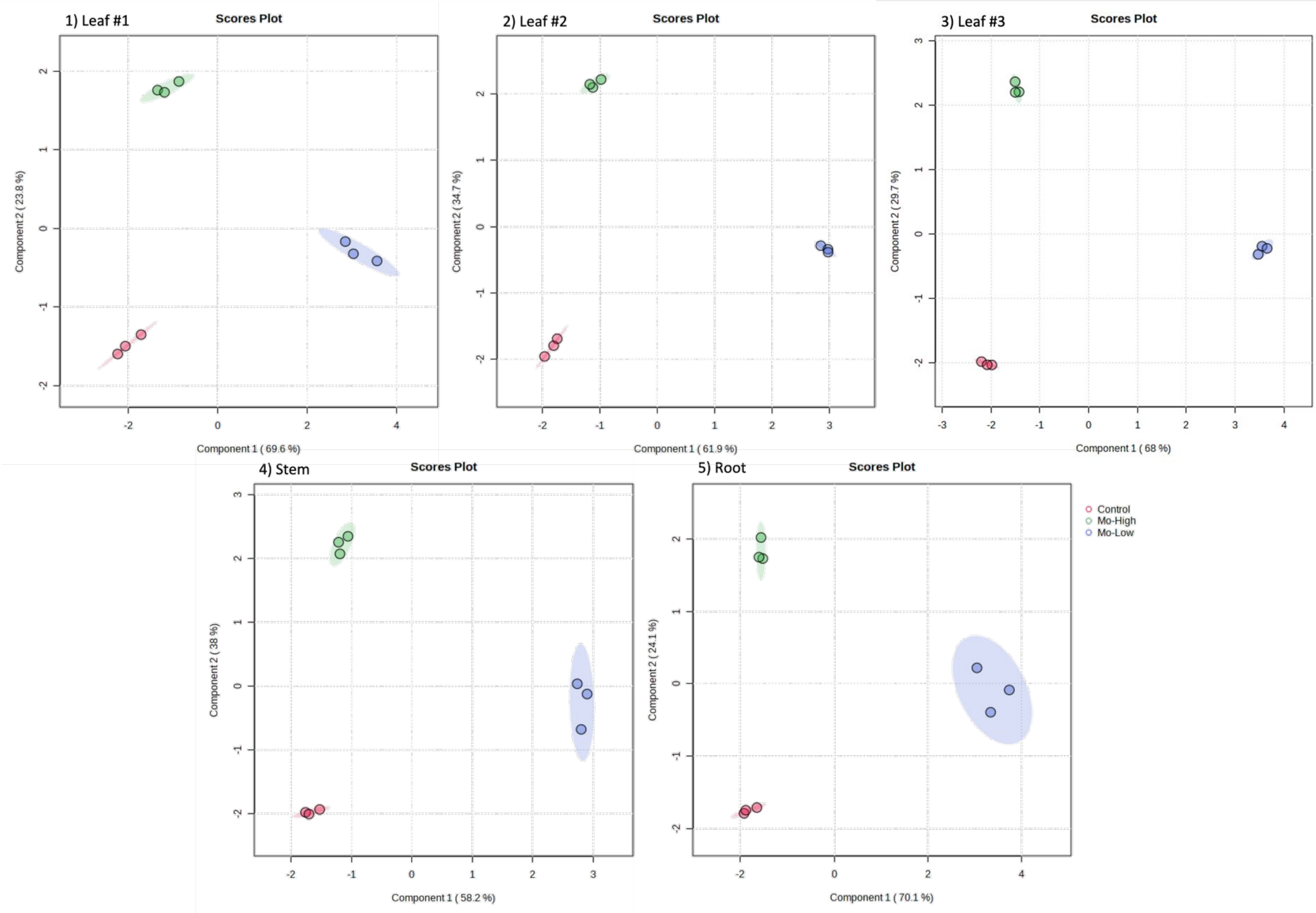

Figure S7. Partial Least Squares Discriminant Analysis (PLS-DA) of metabolite concentrations in each plant tissues with Mo exposure through root at high dose vs. low dose.

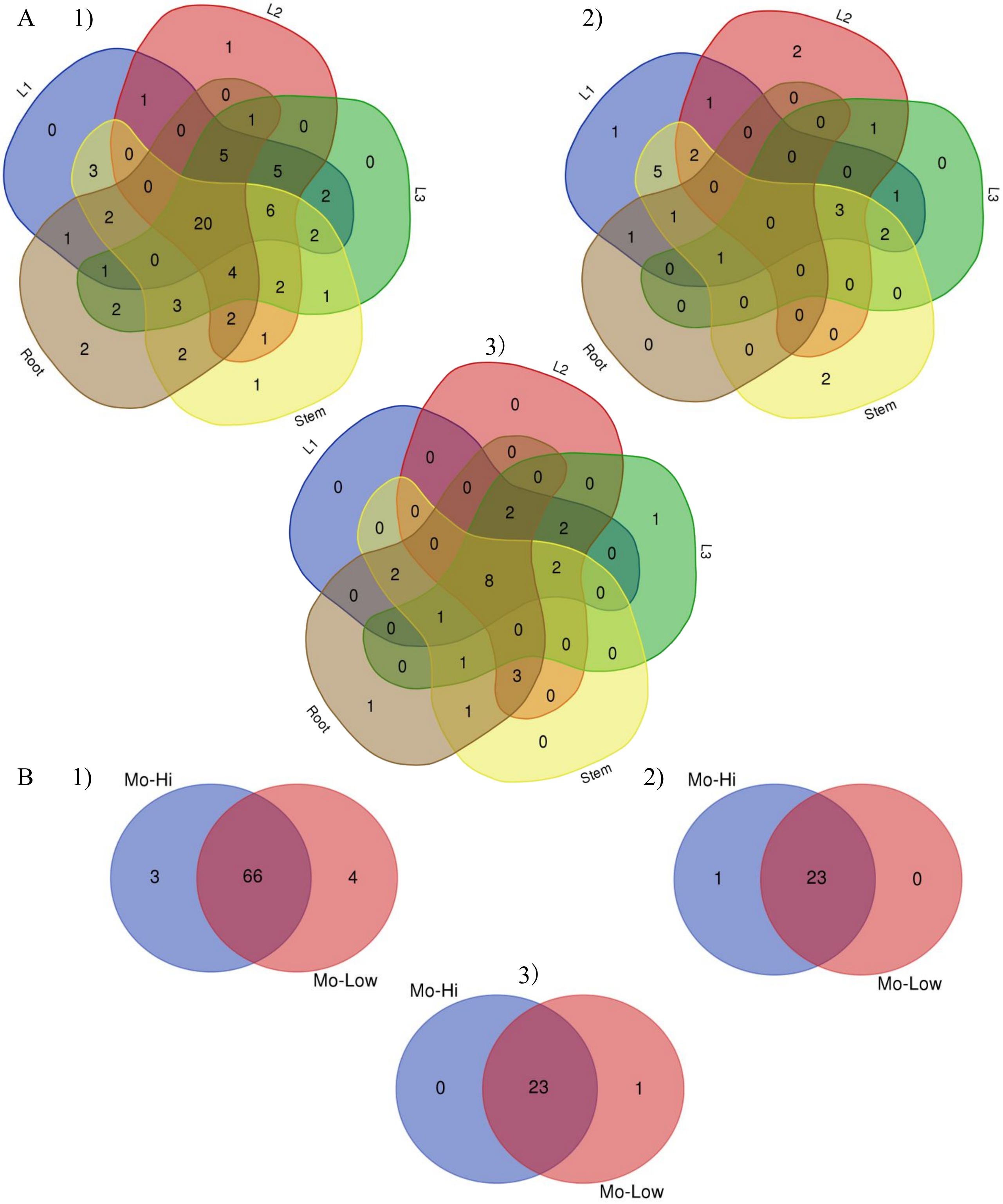

Figure S8. Venn diagram of A) tissue specific distribution for 1)70 responsive metabolites, 2) 23 responsive proteins and 3) 24 perturbed pathways in wheat with low Mo exposure through root;

B) 1) responsive metabolites, 2) responsive proteins and 3) perturbed pathways in wheat with high Mo vs. low Mo exposure through root.

A

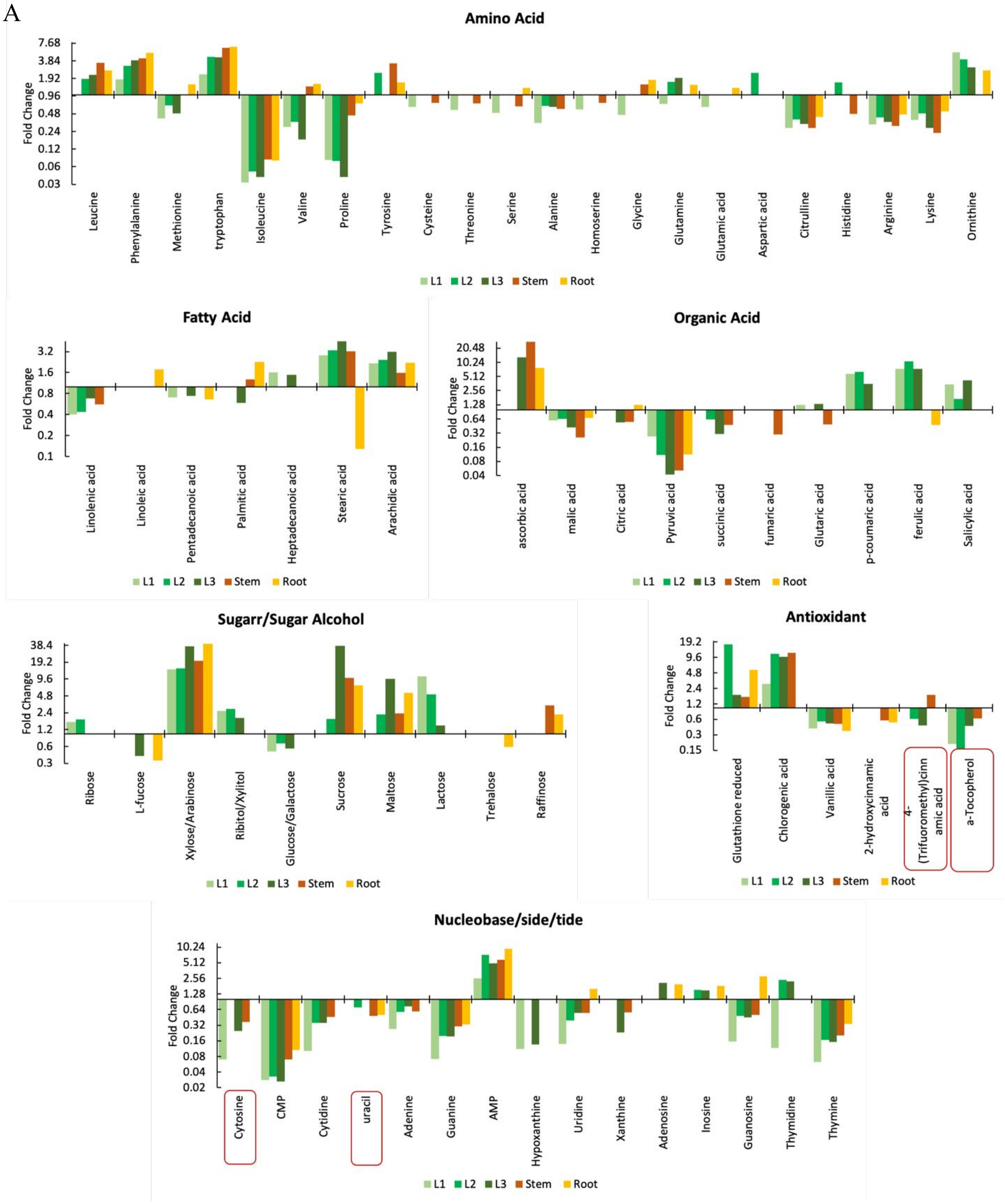

Amino Acid
Fold Change
7.68
3.84
1.92
0.96
0.48
0.24
0.12
0.06
0.03
Leucine
Phenylalanine
Methionine
tryptophan
Isoleucine
Valine
Proline
Tyrosine
Cysteine
Threonine
Serine
Alanine
Homoserine
Glycine
Glutamine
Glutamic acid
Aspartic acid
Citrulline
Histidine
Arginine
Lysine
Ornithine
L1 L2 L3 Stem Root
Fatty Acid
Fold Change
3.2
1.6
0.8
0.4
0.2
0.1
Linolenic acid
Linoleic acid
Pentadecanoic acid
Palmitic acid
Heptadecanoic acid
Stearic acid
Arachidic acid
L1 L2 L3 Stem Root
Organic Acid
Fold Change
20.48
10.24
5.12
2.56
1.28
0.64
0.32
0.16
0.08
0.04
ascorbic acid
malic acid
Citric acid
Pyruvic acid
succinic acid
fumaric acid
Glutaric acid
p-coumaric acid
ferulic acid
Salicylic acid
L1 L2 L3 Stem Root
Sugarr/Sugar Alcohol
Fold Change
38.4
19.2
9.6
4.8
2.4
1.2
0.6
0.3
Ribose
L-fucose
Xylose/Arabinose
Ribitol/Xylitol
Glucose/Galactose
Sucrose
Maltose
Lactose
Trehalose
Raffinose
L1 L2 L3 Stem Root
Antioxidant
Fold Change
19.2
9.6
4.8
2.4
1.2
0.6
0.3
0.15
Glutathione reduced
Chlorogenic acid
Vanillic acid
2-hydroxycinnamic acid
4-(Trifluoromethyl)cinnamic acid
a-Tocopherol
L1 L2 L3 Stem Root
Nucleobase/side/tide
Fold Change
10.24
5.12
2.56
1.28
0.64
0.32
0.16
0.08
0.04
0.02
Cytosine
CMP
Cytidine
uracil
Adenine
Guanine
AMP
Hypoxanthine
Uridine
Xanthine
Adenosine
Inosine
Guanosine
Thymidine
Thymine
L1 L2 L3 Stem Root

B

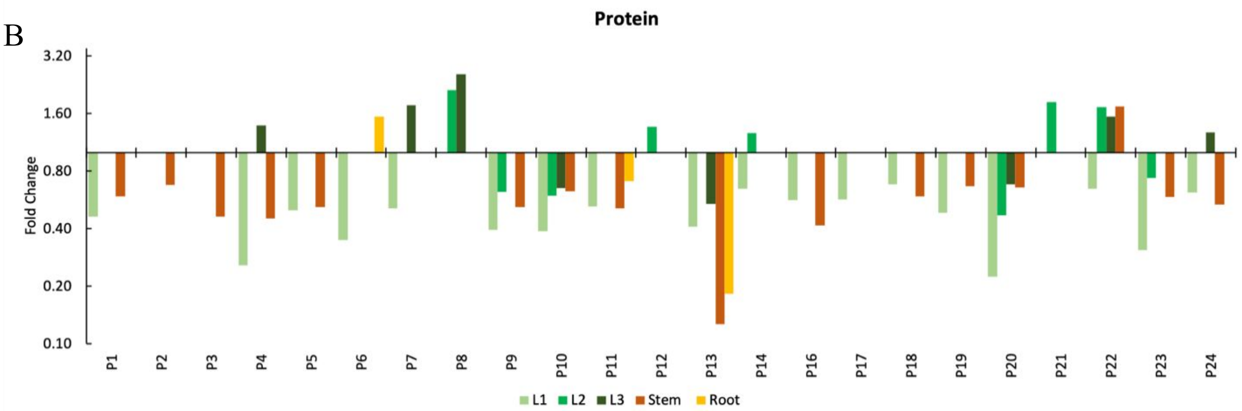

Figure S9. Fold change bar plots of a) 70 responsive metabolites (grouped by metabolite classes) and B) 23 responsive proteins in different plant tissues with Mo exposure at low concentration through Root.

Table S4. Joint-pathway analysis results for Cu exposure. Total is the total number of metabolites in the pathway; hit is the actually matched number of responsive metabolites; bold font means metabolite or protein only involved in perturbed pathways through root exposure; underline font means metabolite or protein only involved in perturbed pathways through leaf exposure.

Pathways	Pathway Class	Total	Cu Exposure							
			Root exposure			Leaf exposure			Metabolites	Proteins
			Hit	Impact	Tissue	Hit	Impact	Tissue		
Alanine, aspartate and glutamate metabolism	Amino acid metabolism	22	5	0.20	L2	3	0.64	L2, L3, S	**Alanine**; Aspartic acid; Glutamine; Glutamic acid; **Fumaric acid**; **Pyruvic acid**; **Succinic acid**	**P13**; **P15**
Arginine and proline metabolism	Amino acid metabolism	34	3	0.27	L1, R	4	0.34	L1, L2, L3	Arginine; Proline; Glutamic acid; Ornithine	P4
Arginine biosynthesis	Amino acid metabolism	18	5	0.31	L1, L2, R	6	0.40	L1, L2, L3	Arginine; Aspartic acid; Citrulline; Ornithine; **Fumaric acid**; Glutamine; Glutamic acid	**P13**; **P15**
Cysteine and methionine metabolism	Amino acid metabolism	46	5	0.19	L1	5	0.18	L1, L2	Aspartic acid; Serine; Methionine; Cysteine; Homoserine; **Pyruvic acid**	P1; P2; P3; **P22**
Glutathione metabolism	Amino acid metabolism	26	-	-	-	4	0.48	L1	Glutathione; Glycine; Glutamic acid; Cysteine	-
Glycine, serine and threonine metabolism	Amino acid metabolism	33	6	0.58	L1, L2, S, R	6	0.58	L1, L2, L3, R	Aspartic acid; Serine; Glycine; Threonine; Homoserine; **Pyruvic acid**; Tryptophan	**P15**
Phenylalanine metabolism	Amino acid metabolism	11	1	0.47	L1, L2, L3	1	0.47	L1, L2, L3	Phenylalanine	-
Tryptophan metabolism	Amino acid metabolism	28	1	0.12	L1, L2	1	0.12	L1, L2	Tryptophan	P4; P21
Tyrosine metabolism	Amino acid metabolism	16	3	0.22	L1, R	1	0.11	L1, L2, L3	Tyrosine; **Fumaric acid**; **Pyruvic acid**	-
Valine, leucine and isoleucine biosynthesis	Amino acid metabolism	22	5	0.11	R	-	-	-	**Threonine; Leucine; Pyruvic acid; Isoleucine; Valine**	-
Biosynthesis of secondary metabolites - unclassified	Biosynthesis of secondary metabolites	5	-	-	-	1	1.00	R	p-coumaric acid	-
Isoquinoline alkaloid biosynthesis	Biosynthesis of secondary metabolites	6	1	0.50	L1	1	0.50	L1, L2, L3	Tyrosine	-
Phenylpropanoid biosynthesis	Biosynthesis of secondary metabolites	46	-	-	-	4	0.10	-	Ferulic acid; Chlorogenic acid; Phenylalanine; p-coumaric acid	-
Stilbenoid, diarylheptanoid and gingerol biosynthesis	Biosynthesis of secondary metabolites	8	1	0.13	L1, L2, L3, S	1	0.13	L3, S	Chlorogenic acid	-
Citrate cycle (TCA cycle)	Carbohydrate metabolism	20	4	0.22	R	-	-	-	**Malic acid**; **Succinic acid**; **Pyruvic acid**; **Fumaric acid**	**P22**; P23
Galactose metabolism	Carbohydrate metabolism	27	-	-	-	2	0.12	L1, L3	Raffinose; Sucrose	**P6**
Glycolysis / Gluconeogenesis	Carbohydrate metabolism	26	2	0.12	R	-	-	-	**Pyruvic acid**; **Lactic acid**	P4; P5; **P17**
Glyoxylate and dicarboxylate metabolism	Carbohydrate metabolism	29	5	0.18	L1, R	4	0.17	L1, L2, L3, R	Serine; **Malic acid**; Glycine; Glutamine; **Succinic acid**; Glutamic acid	P14; **P15**; P21; **P22**; P23
Pyruvate metabolism	Carbohydrate metabolism	22	4	0.32	R	-	-	-	**Malic acid**; **Pyruvic acid**; **Lactic acid**; **Fumaric acid**	P4; **P22**
alpha-Linolenic acid metabolism	Lipid metabolism	28	1	0.11	L1	1	0.11	L1, L2, L3	Linolenic acid	-
Linoleic acid metabolism	Lipid metabolism	4	1	1.00	R	1	1.00	L1, L2, L3	Linoleic acid	-
Purine metabolism	Nucleotide metabolism	63	-	-	-	-	-	-	-	-
Aminoacyl-tRNA biosynthesis	Translation	46	17	0.11	L1, L2, S	18	0.11	L1, L2, L3	Aspartic acid; Histidine; Phenylalanine; Arginine; Glutamine; Cysteine; Glycine; Serine; Methionine; Valine; **Alanine**; Lysine; Isoleucine; Leucine; Threonine; Tryptophan; Tyrosine; Proline; Glutamic acid	-

Table S5. Joint-pathway analysis results for Mo exposure. Total is the total number of metabolites in the pathway; hit is the actually matched number of responsive metabolites; bold font means metabolite or protein only involved in perturbed pathways through root exposure; underline font means metabolite or protein only involved in perturbed pathways through leaf exposure.

Pathways	Pathway Class	Total	Mo Exposure							
			Root exposure			Leaf exposure			Metabolites	Proteins
			Hit	Impact	Tissue	Hit	Impact	Tissue		
Alanine, aspartate and glutamate metabolism	Amino acid metabolism	22	7	0.65	L1, L2, L3, S, R	5	0.52	L1, S, R	**Aspartic acid**; Alanine; Glutamine; Glutamic acid; Fumaric acid; **Pyruvic acid**; Succinic acid	**P13**; P15
Arginine and proline metabolism	Amino acid metabolism	34	4	0.34	L1, L2, L3, S, R	4	0.34	L1, L2, L3, R	Arginine; Proline; Glutamic acid; Ornithine;	**P4**
Arginine biosynthesis	Amino acid metabolism	18	7	0.40	L1, L2, L3, S, R	6	0.40	L1, L2, L3, R	Glutamic acid; Arginine; Citrulline; **Aspartic acid**; Ornithine; Fumaric acid; Glutamine	**P13**; P15
Cysteine and methionine metabolism	Amino acid metabolism	46	6	0.19	L1, L2, L3, S, R	2	0.18	L1, R	**Serine**; Methionine; Cysteine; **Homoserine**; **Aspartic acid**; **Pyruvic acid**	**P1**; P2; P3; P22
Glutathione metabolism	Amino acid metabolism	26	4	0.48	L3, S, R	4	0.48	L1, L3	Glutathione; Glycine; Glutamic acid; Cysteine;	-
Glycine, serine and threonine metabolism	Amino acid metabolism	33	7	0.58	L1, L2, L3, S, R	2	0.21	L2	**Serine**; Glycine; **Aspartic acid**; **Threonine**; **Homoserine**; **Pyruvic acid**; Tryptophan	P15
Phenylalanine metabolism	Amino acid metabolism	11	1	0.47	L1, L2, L3, S, R	1	0.47	L1, L2, R	Phenylalanine	-
Tryptophan metabolism	Amino acid metabolism	28	1	0.12	L1, L2, L3, S, R	1	0.12	L3	Tryptophan	**P4**; **P21**
Tyrosine metabolism	Amino acid metabolism	16	3	0.22	L1, L2, L3, S, R	1	0.11	R	**Tyrosine**; Fumaric acid; **Pyruvic acid**	-
Valine, leucine and isoleucine biosynthesis	Amino acid metabolism	22	5	0.11	L2, S, R	-	-	-	**Threonine**; Leucine; **Pyruvic acid**; Isoleucine; Valine	-
Biosynthesis of secondary metabolites - unclassified	Biosynthesis of secondary metabolites	5	1	1.00	L1, L2, L3, S, R	1	1.00	L1, L2, L3, R	p-coumaric acid	-
Isoquinoline alkaloid biosynthesis	Biosynthesis of secondary metabolites	6	1	0.50	L2, L3, S, R	-	-	-	**Tyrosine**	-
Phenylpropanoid biosynthesis	Biosynthesis of secondary metabolites	46	4	0.10	L2, L3, S, R	4	0.10	L1, L2, L3, R	Ferulic acid; Chlorogenic acid; Phenylalanine; p-coumaric acid;	-
Stilbenoid, diarylheptanoid and gingerol biosynthesis	Biosynthesis of secondary metabolites	8	1	0.13	L2, L3, S, R	1	0.13	L1, L2, L3, S, R	Chlorogenic acid	-
Citrate cycle (TCA cycle)	Carbohydrate metabolism	20	5	0.22	L1, L2, L3, S	-	-	-	**Malic acid**; Succinic acid; **Citric acid**; **Pyruvic acid**; Fumaric acid	P22; **P23**
Galactose metabolism	Carbohydrate metabolism	27	3	0.12	L2, L3, S	2	0.12	L1,S, R	Raffinose; Sucrose; **Galactose**	**P6**
Glycolysis / Gluconeogenesis	Carbohydrate metabolism	26	3	0.12	L1, L2, S, R	-	-	-	**Pyruvic acid**; **Lactic acid**; **Glucose**	**P4**; P5; P17; P18
Glyoxylate and dicarboxylate metabolism	Carbohydrate metabolism	29	7	0.24	L2, L3, S, R	4	0.15	L2	**Serine**; **Malic acid**; **Citric acid**; Glycine; Glutamic acid; Glutamine; Succinic acid	**P14**; P15; **P21**; P22; **P23**
Pyruvate metabolism	Carbohydrate metabolism	22	4	0.32	L1, L2, L3, S, R	-	-	-	**Malic acid**; **Pyruvic acid**; **Lactic acid**; Fumaric acid	**P4**; P22
alpha-Linolenic acid metabolism	Lipid metabolism	28	1	0.11	R	-	-	-	**Linolenic acid**	-
Linoleic acid metabolism	Lipid metabolism	4	1	1.00	L1, L2, L3, S, R	-	-	-	**Linoleic acid**	P8
Purine metabolism	Nucleotide metabolism	63	9	0.11	-	7	0.10	L2	Xanthine; Glutamine; AMP; **Adenosine**; Hypoxanthine; Guanine; **Adenine**; inosine; Guanosine	-
Aminoacyl-tRNA biosynthesis	Translation	46	19	0.11	L1, L2, L3, S, R	-	-	-	Histidine; Phenylalanine; Arginine; Glutamine; Cysteine; Glycine; **Aspartic acid**; **Serine**; Methionine; Valine; Alanine; Lysine; Isoleucine; Leucine; **Threonine**; Tryptophan; **Tyrosine**; Proline; Glutamic acid	-

Table S6. Joint-pathway analysis results for Mo exposure with high vs. low dose. (Bold means only involved in pathways with high exposure; Underline means only involved in pathways with low dose exposure)

Pathways	Pathway Class	Total	Mo Exposure through root							
			High Dose			Low Dose			Metabolites	Proteins
			Hit	Impact	Tissue	Hit	Impact	Tissue		
Alanine, aspartate and glutamate metabolism	Amino acid metabolism	22	7	0.65	L1, L2, L3, S, R	7	0.65	L1, L2, L3, R	Aspartic acid; Alanine; Glutamine; Glutamic acid; Fumaric acid; Pyruvic acid; Succinic acid	P13; **P15**
Arginine and proline metabolism	Amino acid metabolism	34	4	0.34	L1, L2, L3, S, R	4	0.34	L1, L2, L3, S, R	Arginine; Proline; Glutamic acid; Ornithine;	P4
Arginine biosynthesis	Amino acid metabolism	18	7	0.40	L1, L2, L3, S, R	7	0.40	L1, L2, L3, S, R	Glutamic acid; Arginine; Citrulline; Aspartic acid; Ornithine; Fumaric acid; Glutamine	P13; **P15**
Cysteine and methionine metabolism	Amino acid metabolism	46	6	0.19	L1, L2, L3, S, R	6	0.19	L1, L2, L3, R	Serine; Methionine; Cysteine; Homoserine; Aspartic acid; Pyruvic acid;	P1; P2; P3; P22
Glutathione metabolism	Amino acid metabolism	26	4	0.48	L3, S, R	4	0.48	L1, L2, L3, S, R	Glutathione; Glycine; Glutamic acid; Cysteine;	-
Glycine, serine and threonine metabolism	Amino acid metabolism	33	7	0.58	L1, L2, L3, S, R	7	0.58	L1, S, R	Serine; Glycine; Aspartic acid; Threonine; Homoserine; Pyruvic acid; Tryptophan	**P15**
Phenylalanine metabolism	Amino acid metabolism	11	1	0.47	L1, L2, L3, S, R	1	0.47	L1, L2, L3, S, R	Phenylalanine	-
Tryptophan metabolism	Amino acid metabolism	28	1	0.12	L1, L2, L3, S, R	1	0.12	L1, L2, L3, S, R	Tryptophan	P4; P21
Tyrosine metabolism	Amino acid metabolism	16	3	0.22	L1, L2, L3, S, R	3	0.22	L2, S, R	Tyrosine; Fumaric acid; Pyruvic acid	-
Valine, leucine and isoleucine biosynthesis	Amino acid metabolism	22	5	0.11	L2, S, R	5	0.11	L1, L2, L3, S, R	Threonine; Leucine; Pyruvic acid; Isoleucine; Valine;	-
Biosynthesis of secondary metabolites - unclassified	Biosynthesis of secondary metabolites	5	1	1.00	L1, L2, L3, S, R	1	1.00	L1, L2, L3	p-coumaric acid	-
Isoquinoline alkaloid biosynthesis	Biosynthesis of secondary metabolites	6	1	0.50	L2, L3, S, R	1	0.50	L2, S, R	Tyrosine	-
Phenylpropanoid biosynthesis	Biosynthesis of secondary metabolites	46	4	0.10	L2, L3, S, R	4	0.10	L1, L2, L3	Ferulic acid; Chlorogenic acid; Phenylalanine; p-coumaric acid;	-
Stilbenoid, diarylheptanoid and gingerol biosynthesis	Biosynthesis of secondary metabolites	8	1	0.13	L2, L3, S, R	1	0.13	L1, L2, L3, S	Chlorogenic acid	-
Citrate cycle (TCA cycle)	Carbohydrate metabolism	20	5	0.22	L1, L2, L3, S	5	0.22	L3, S, R	Malic acid; Succinic acid; Citric acid; Pyruvic acid; Fumaric acid;	P22; P23
Galactose metabolism	Carbohydrate metabolism	27	3	0.12	L2, L3, S	3	0.12	S, R	Raffinose; Sucrose; Galactose	P6
Glycolysis / Gluconeogenesis	Carbohydrate metabolism	26	3	0.12	L1, L2, S, R	2	0.12	L1, L2, L3, S, R	Pyruvic acid; **Lactic acid**; Glucose	P4; P5; P17; P18
Glyoxylate and dicarboxylate metabolism	Carbohydrate metabolism	29	7	0.24	L2, L3, S, R	7	0.24	L1, S, R	Serine; Malic acid; Citric acid; Glycine; Glutamic acid; Glutamine; Succinic acid	P14; **P15**; P21; P22; P23
Pyruvate metabolism	Carbohydrate metabolism	22	4	0.32	L1, L2, L3, S, R	3	0.32	L1, L2, L3, S, R	Malic acid; Pyruvic acid; **Lactic acid**; Fumaric acid	P4; P22
alpha-Linolenic acid metabolism	Lipid metabolism	28	1	0.11	R	1	0.11	L1, L2, L3, S	Linolenic acid	-
Linoleic acid metabolism	Lipid metabolism	4	1	1.00	L1, L2, L3, S, R	1	1.00	R	Linoleic acid	P8
Purine metabolism	Nucleotide metabolism	63	9	0.11	-	9	0.11	L3	Xanthine; Glutamine; AMP; Adenosine; Hypoxanthine; Guanine; Adenine; inosine; Guanosine	-
Pyrimidine metabolism	Nucleotide metabolism	38	-	-	-	7	0.13	L2, S, R	Glutamine; Uridine; CMP; Cytidine; Thymidine; Thymine; Uracil	-
Aminoacyl-tRNA biosynthesis	Translation	46	19	0.11	L1, L2, L3, S, R	19	0.11	L1, S, R	Histidine; Phenylalanine; Arginine; Glutamine; Cysteine; Glycine; Aspartic acid; Serine; Methionine; Valine; Alanine; Lysine; Isoleucine; Leucine; Threonine; Tryptophan; Tyrosine; Proline; Glutamic acid	-
